# Public support in the United States for global equity in vaccine pricing

**DOI:** 10.1038/s41598-022-13172-7

**Published:** 2022-05-27

**Authors:** Yee Chan, Gaurav Datt, Asadul Islam, Birendra Rai, Liang C. Wang

**Affiliations:** 1 Department of Economics, Monash University, Clayton, VIC 3800 Australia; 2 Centre for Development Economics and Sustainability, Monash University, Caulfield East, VIC 3145 Australia

**Keywords:** Health policy, Public health, Health care economics

## Abstract

Global vaccine prices that are tiered across countries, equitable for poorer countries, and profitable for manufacturers (TEP) can promote global vaccine equity but its implementation may require political will and public support in rich countries. A survey experiment with a demographically representative sample of US adults was conducted between April and May 2021 to investigate public support for TEP and the likelihood of collective agreement on TEP relative to alternative global vaccine pricing strategies. The experiment varied vaccine cost and provision of information about the importance of equity and profitability considerations in global vaccine pricing across eight treatment conditions. TEP of low-cost vaccines received less support than TEP of high-cost vaccines, but TEP received more public support than any alternative pricing strategy. Information about equity and profitability considerations increased support for TEP of low-cost vaccines. TEP was also the most likely pricing strategy to achieve collective agreement among participants across all treatments.

## Introduction

The novel coronavirus disease (COVID-19) pandemic has intensified efforts to address global vaccine inequity^1–3^. International health agencies, governments, philanthropists, and the private sector have joined the COVAX facility to facilitate timely and global equitable access to COVID-19 vaccines^4^. The main aim of the COVAX facility is ensuring that a country’s ability to pay is not a barrier to access vaccines by negotiating pooled procurement agreements with manufacturers. An important part of these negotiations is tiered pricing—charging lower prices to poorer countries that lack economic resources but have a high disease burden, while charging richer countries higher prices for the same vaccine to ensure profitability for manufacturers^5,6^.

Given the extent of poverty and burden of vaccine-preventable diseases in many poorer countries, there have been calls for global vaccine pricing that is tiered across countries, equitable for poorer countries, and profitable for manufacturers (TEP)^5,7–9^. Under TEP, equity considerations imply prices must be affordable for poorer countries, which requires prices charged to poorer countries should not be significantly higher than the production cost of vaccines^10–13^. However, this inevitably requires richer countries to pay a significant premium above production costs.

The calls for richer countries to donate excess vaccines to poorer countries in the context of the COVID-19 pandemic are broadly consistent with the spirit of TEP. However, pledges about the quantity and timing of donation of vaccines are not binding, and the choice of recipient countries may be based on geo-political considerations rather than global health considerations^14^. The 2009 H1N1 pandemic and the COVID-19 pandemic in fact suggest that vaccine hoarding by rich countries typically precedes the actual donation of vaccines to poor countries, a pattern of decision-making that is neither surprising nor necessarily unethical^15,16^. If anything, it underscores the point that the issue of global vaccine equity calls for thinking about ways to reduce vaccine-preventable deaths in general. The significance of TEP lies in that it provides a structured way to further the goal of vaccine equity without relying too much on the benevolence of the political leaders in richer countries.

TEP, however, faces multiple challenges as it seeks to balance equity and profitability. Manufacturers may be reluctant to support TEP due to fear of leakage or arbitrage, which reduces profits and threatens intellectual property rights^10–13,17^. TEP may face opposition from the general public in richer countries, who may perceive it unfair and exploitative, and be concerned about higher domestic vaccine prices or additional taxes^18,19^. The extent to which public support for TEP in developed countries can translate into actual modifications in global vaccine pricing will, however, depend on many factors, such as governmental purview in vaccine pricing, and the willingness or capability of relevant entities such as GAVI to respond to public views. Existing studies on TEP have largely focused on highlighting its implications for improved access to vaccines for poorer countries and incentives to manufacturers for development of vaccines^5–7,10–13,17–20^. However, there is a paucity of empirical evidence about the support in high-income countries for TEP.

Support for TEP may depend on vaccine cost. For example, higher costs will likely lead to a higher vaccine price negotiated between a government and a pharmaceutical company. People will either directly face a higher retail price for the vaccine or indirectly bear a greater tax burden if prices are not directly passed down to the consumer. However, as higher cost also implies lower affordability for poor countries, public support in high-income countries for TEP may increase if most of their citizens appreciate that during a global pandemic, “no one is safe until everyone is safe”. Consequently, it is possible that public support for TEP is relatively lower for low-cost vaccines, especially those that are typically used to prevent diseases that disproportionately burden poorer countries (e.g., tuberculosis, meningitis and measles)^21,22^.

Information may also influence support for TEP. For example, opposition to TEP due to price gouging concerns may be driven by lack of knowledge about poverty and the disease burden in poorer countries^18,19^. Similarly, lack of awareness regarding the importance of equity and profitability considerations in global vaccine pricing may also undermine support for TEP. Therefore, provision of information, whether it is based on facts, arguments, or both facts and arguments, can potentially be used to enhance public support for TEP.

The purpose of the current study was to quantify the extent of public support in the US for TEP relative to other pricing strategies, and how public support may vary depending on vaccine cost and provision of information. We examined public support for TEP in two different ways: at the individual level, and at the collective level. Participants were asked to choose vaccine prices across different income-based country groups, and whether manufacturers should make profits in a hypothetical scenario. These hypothetical scenarios abstract away from many important aspects of vaccine pricing that entities like GAVI and COVAX have to grapple with in negotiations with pharmaceutical companies. However, the decisions made by participants allowed us to investigate our two core questions: the effects of vaccine cost and information about equity and profitability considerations on (i) individual-level support for TEP, and on (ii) the likelihood of collective-agreement on TEP.

## Methods

### Study sample and procedure

The cross-sectional survey experiment was conducted in the US in April and May 2021. The experiment was programmed in Qualtrics and conducted via Prolific, a UK-based online research platform. The final sample included 803 adult participants living in the US who represented the adult US population aged 18 and over in terms of age, sex, and race (see Supplementary Table S2).

### Ethics declaration

The survey experiment (Project ID 27,264) was approved by the Monash University Human Research Ethics Committee (MUHREC). The survey experiment was performed in accordance with MUHREC’s guidelines and regulations. Informed consent was obtained from all participants.

### Design of experiment

Participants were randomly assigned to one of eight treatment groups (two vaccine cost conditions × four information conditions). Each treatment group had roughly 100 participants. The experiment consisted of several parts (see Supplementary information). After providing informed consent, participants answered a pre-experiment survey containing two questions about their attitudes towards inequality, five questions about their experiences during the COVID-19 pandemic, and a total of seven questions about their baseline knowledge of factors relevant to vaccine pricing (e.g., whether lower-middle income countries are eligible for GAVI support to access vaccines). Since the study was conducted during the COVID-19 pandemic, it was important to check whether exposure to COVID-19 and attitudes towards vaccination were balanced across all groups.

Participants then read information about the basic concepts and choice situations in the experiment, completed a quiz designed to assess their understanding of the experimental instructions, read information in their randomly assigned condition, completed the tasks measuring individual and collective-level support for TEP relative to other pricing strategies, and provided basic socio-demographic information. Participants across the eight treatment groups were balanced in terms of exposure to Covid-19 and attitudes towards vaccination as elicited in the pre-experiment survey, as well as their demographic characteristics. Further, baseline knowledge of factors relevant to vaccine pricing was generally poor and balanced across the eight treatment groups (see Supplementary Table S2).

### Cost and information conditions

To examine how vaccine cost and information related to equity and profitability considerations interact, we chose two cost conditions and four information conditions. We needed at least two cost conditions for a meaningful study. The low-cost condition was set at USD 10 per dose, while the high-cost was set at USD 50 per dose. These cost levels are based on price data reported by WHO, where the highest price paid by poorer countries was about USD 10 per dose and the highest price paid by richer countries was about USD 50 per dose^23^. Alternatively, the low-cost conditions may be viewed as reflecting as scenarios where intellectual property rights have been temporarily suspended or permanently expired, or the availability of generic brands at later stages in the product cycle^24^. For both cost conditions, the production (marginal) and pre-production (fixed) cost were set at 30% and 70%, to reflect the relatively high costs of vaccine R&D^25–27^.

The four information conditions are: Arguments, Facts, Arguments + Facts, and NoInfo. Our choice of the nature and number of information conditions was guided by potential heterogeneity in what it takes to influence people’s opinion: empirical facts, conceptual arguments, or both. The Arguments condition provided arguments (without supporting facts) in favor of ensuring both affordability for poorer countries and profitability for manufacturers (Table 1). The advantage of these arguments is that they are relatively simple and can potentially help individuals reason the implications of different decisions. The Facts condition provided statistics about the four country groups relevant to equity and profitability considerations in global vaccine pricing (Table 1). Knowledge of such facts could be useful in understanding the relevance and importance of importance of equity and profitability considerations in global vaccine pricing. The Arguments + Facts condition combined the Arguments and Facts conditions by using the statistics from the Facts condition to justify the arguments in the Arguments condition. Participants in the NoInfo condition were not provided any arguments or facts. The four information conditions along with the two cost conditions constitute the total eight treatment groups in our study. This led us to select roughly 100 participants per treatment group because in order to generate a demographically representative sample of the adult US population within the study period.

**Table 1:** Information provided in the Arguments condition and Facts condition.

Arguments	Description
Equity	Vaccine price should be affordable for all countries and based on a country’s ability to pay
	Vaccines should be available to countries that need them the most as quickly as possible
Profitability	Firms should have sufficient incentive to research, develop, innovate and supply vaccines

Facts	Description
Equity	Average income per person per day by country group (USD)
	Share of the world’s population by country group (%)
	Share of the world’s extremely poor population by country group (%)
	Share of the world’s vaccine-preventable deaths by country group for selected diseases (%)
	Eligibility for GAVI support to access vaccines by country group (Yes/No)
Profitability	Average cost of R&D for selected pharmaceutical products (USD)
	Average time for development for selected pharmaceutical products (years)
	Probability of development and regulatory approval for selected pharmaceutical products (%)
	Global pharmaceutical market value for selected pharmaceutical products (USD)

The full text of each fact and each argument is included in the Supplementary information section. Factual information was provided to participants in tables of statistics. Selected diseases used in “Share of the world’s vaccine-preventable deaths by country group (%)” were: Tuberculosis, Whooping cough, Tetanus, Measles, Varicella and herpes zoster, Acute hepatitis A, Acute hepatitis B, Typhoid fever, Pneumococcal meningitis, H influenzae type B meningitis, Meningococcal meningitis, Diphtheria, Dengue, and Yellow fever. Selected pharmaceutical products for profitability-related facts were: vaccines, cardiovascular disease drugs and cancer drugs.

### Choice scenario

Participants were presented with a hypothetical scenario where, in a global pandemic, a firm with the ability to supply the whole world had developed a safe and effective vaccine that had been approved by health authorities. This framing was used to encourage participants to focus on the tension between equity and profitability, and minimize concerns about vaccine safety, effectiveness, or supply. The instructions to the participants did not explicitly mention this vaccine was for COVID-19. However, participants are likely to have been thinking about vaccines for COVID-19 while making their choices given the study was conducted during the COVID-19 pandemic. We therefore took care to ensure participants were randomly assigned to the treatment groups and checked for balance in demographic characteristics and baseline knowledge about factors relevant to global vaccine pricing. Thus, any priming due to COVID-19 is unlikely to impact the validity of our findings.

Participants chose the price per dose of vaccine that the firm should charge for each of the four country groups according to the income classification by World Bank: low-, lower-middle, upper-middle, and high-income countries^28^. For each country group, participants were asked to choose one out of the following six price categories (from lowest to highest): (1) below marginal cost, (2) equal to marginal cost, (3) above marginal cost but significantly less than total cost, (4) significantly above marginal cost but less than total cost, (5) equal to total cost, (6) above total cost. It was emphasized that prices refer to prices paid by a country to the manufacturer, and that governments may choose to offer vaccines to their citizens for free. After choosing the vaccine prices for all four country groups, participants answered a question about whether the firm should make an “overall profit”. They could respond either “Yes”, “No”, or “Unsure”. Participants answered these questions in an individual task measuring the individual-level (personal) views of participants.

After the individual task, participants answered the same questions in a coordination task designed to reveal the pricing strategy most likely to achieve collective agreement. For each coordination decision, a participant could earn a bonus of GBP 0.10 if their decision matched the most frequent decision among all participants.

In both the individual task and the coordination task, participants were asked to make the same decisions about vaccine pricing in relation to the same hypothetical scenario. However, there is a fundamental difference between the two tasks in terms of what they encourage participants to think about before making their choices. A participant does not need to think about the views of other participants about vaccine pricing while responding in the individual task, as it is designed to elicit an individual’s personal views and preferences. In contrast, the coordination task is designed to encourage participants to think about the views of others. Each participant knows they will receive a bonus payment if their reported pricing choices match with the most frequent pricing choices among all the participants. This feature likely encourages participants to think about the views of others, rather than their own personal preferences. The choices made in the coordination task reveal what people with potentially different personal preferences are most likely to collective agree upon^29^.

### Outcome measures

Our primary outcome was support for TEP as measured by three components: tiering of prices across countries, equitable prices for poorer countries, and profitability for manufacturers. As tiered pricing requires poorer countries to be charged a lower price than richer countries, a participant was deemed to support tiered pricing if two conditions were met: (i) the prices for low- and lower-middle income countries were lower than the price for upper-middle income countries, (ii) the price for upper-middle income countries was less than or equal to the price for high-income countries. Equitable pricing was defined as prices for low- and lower-middle income countries that were close to the production (marginal) cost, i.e., below marginal cost, equal to marginal cost, or above marginal cost but significantly less than total cost^12,17,19^. Profitable pricing was defined as a participant who responded “Yes” to firms making overall profits.

We coded a participant as supporting TEP only if they supported all three components. Otherwise, we classified choices as falling within three alternative pricing strategies: support for tiered pricing and equitable pricing but not profitable pricing (TEnotP), support for tiered pricing and profitable pricing but not equitable pricing (TPnotE), and a residual category capturing all other decisions (Other).

### Data analysis

We first used participants’ responses to seven questions about prior familiarity with GAVI, COVAX, and factors relevant to vaccine pricing to assess whether participants had “sufficient baseline knowledge” about factors that are relevant to TEP (defined as score of at least four out of seven). Only 17% of participants had sufficient baseline knowledge. This low level of baseline knowledge is statistically similar across all groups (see Supplementary Table S2).

We examined the effects of an increase in vaccine cost and provision of information by comparing the level of support for a pricing strategy in each treatment group to the LowCost × NoInfo baseline group. We consider the LowCost × NoInfo baseline group as the control group where the participants in this group face a low-cost vaccine and possess a low level of information relevant for TEP. Given the uniformly low fraction of participants with sufficient baseline knowledge in all cost and information conditions, the treatment differences relative to LowCost × NoInfo control group are interpreted as the impact of “increasing vaccine cost” and/or “increasing information” on public support for TEP.

Treatment effects were estimated using multivariate ordinary least squares regression. We included controls for comprehension of experimental instructions, whether participants paid attention during the experiment. In addition, we controlled for whether a participant had sufficient baseline knowledge of factors relevant to vaccine pricing. T-tests were used to evaluate treatment effects and differences between groups. We considered *p* values of 0.10 or less to be significant. Statistical analyses were conducted using STATA SE, version 16.

## Results

We first present the results from combining the data from the three information conditions (Arguments, Facts, Arguments + Facts) to investigate the effect of provision of any information and vaccine cost on individual-level support for each pricing strategy. Thus, the four relevant groups in this analysis will be LowCost × NoInfo as the baseline group, and LowCost × AnyInfo, HighCost × NoInfo, and HighCost × AnyInfo as the three comparison groups.

**Figure 1:**
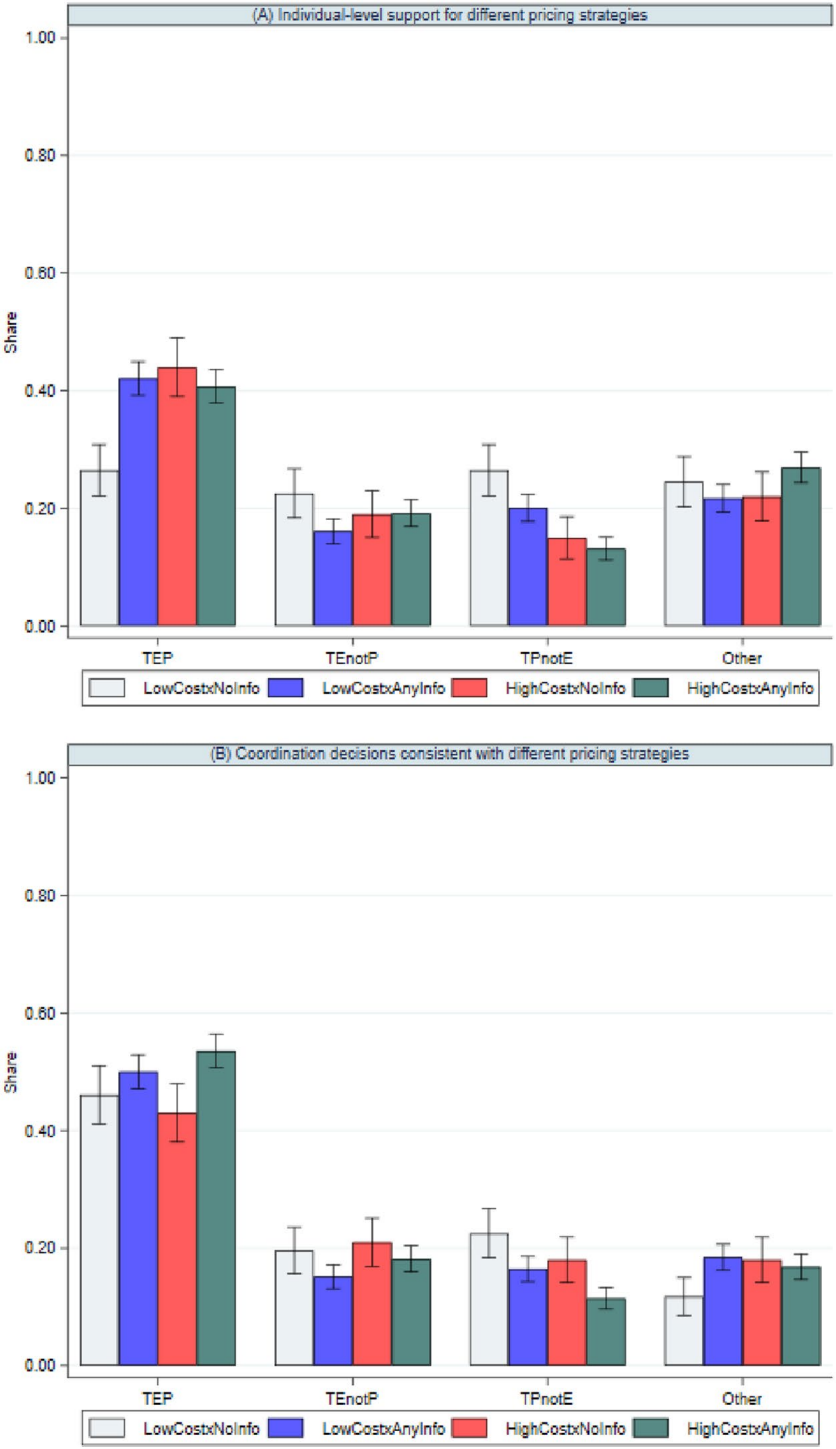
Support for different pricing strategies by vaccine cost and information. *Notes*: Data from all three information conditions—Arguments, Facts, and Arguments + Facts—were pooled into a single “AnyInfo” condition. TEP refers to a pricing strategy that is tiered across countries, equitable for poorer countries, and profitable for manufacturers. TEnotP refers to a tiered pricing strategy that is equitable but not profitable. TPnotE refers to a tiered pricing strategy that is profitable but not equitable. Other refers to any pricing strategy other than TEP, TEnotP, and TPnotE. Error bars represent the mean ± standard errors of the mean.

TEP was generally the most personally preferred pricing strategy out of all pricing strategies (panel A of Fig. 1). TEP received the highest support and more support than any other pricing strategy. The only exception was in the LowCost × NoInfo baseline, where support was similar among the TEP, TEnotP, TPnotE, and Other pricing strategies: 26%, 23%, 26%, and 25% respectively. Information provision was more effective for low-cost vaccines than high-cost vaccines in increasing support for TEP (panel A of Fig. 1). For low-cost vaccines, support for TEP increased from 26% (LowCost × NoInfo baseline) to 42% (LowCost × AnyInfo) as a result of information provision. By contrast, relative to the LowCost × NoInfo baseline, an increase in vaccine cost increased support for TEP (44%, HighCost × NoInfo), but there was no additional impact from information provision (41%, HighCost × AnyInfo).

Given the fundamental difference between the individual task and the coordination task—thinking about one’s personal preferences versus thinking about views of others—the choices made by participants in the two tasks need not necessarily be the same. Nevertheless, we find that TEP was the most frequently chosen pricing strategy when subjects made coordination decisions, regardless of vaccine cost and provision of information (panel B of Fig. 1). The share of coordination decisions consistent with TEP was always greater than the corresponding shares for each alternative pricing strategy, regardless of vaccine cost and information provision. For example, in the LowCost × NoInfo baseline, the share of coordination decisions consistent with TEP was 46%, while the corresponding shares for TEnotP, TPnotE, and Other were far lower, at only 20%, 23%, and 12% respectively. Panel B of Fig. 1 also shows that the share of coordination decisions consistent with TEP was not particularly sensitive to vaccine costs or information provision (ranged from 43 to 58%). These findings suggest that, regardless of vaccine cost and information provision, TEP is the most likely pricing strategy to achieve collective agreement among the participants.

Table 2 reports the disaggregated analysis of the impact of an increase in vaccine cost and/or provision of information about equity and profitability considerations on the extent of support for TEP at the individual level (column 1) and collective level (column 2). The results confirm the findings regarding individual-level support from pooling the data on the three information conditions. For example, column 1 in Table 2 shows individual-level support for TEP of low-cost vaccines increased significantly with the provision of information on arguments (16.7 percentage points; *p* = 0.01), facts (19.0 percentage points; *p* = 0.004), or both facts and arguments (12.8 percentage points; *p* = 0.049). In contrast, column 2 in Table 2 highlights that the cost and information treatments had little effect on the share of coordination decisions consistent with TEP relative to the LowCost × NoInfo baseline, except the provision of information on arguments for high-cost vaccines (13.1 percentage points; *p* = 0.059). Thus, vaccine costs and provision of information influenced support for TEP only at the individual level, but not at the collective level.

**Table 2:** Impact of vaccine cost and information conditions.

	TEP		Conflicted	
	Personal	Coordination	Personal	Coordination
LowCost × Arguments (n = 101)	0.167***	0.060	− 0.181***	− 0.057
	(0.065)	(0.069)	(0.067)	(0.070)
LowCost × Facts (n = 101)	0.190***	0.050	− 0.213***	− 0.023
	(0.065)	(0.070)	(0.067)	(0.070)
LowCost × Arguments + Facts (n = 102)	0.128**	0.019	− 0.129*	− 0.000
	(0.065)	(0.069)	(0.066)	(0.069)
HighCost × NoInfo (n = 100)	0.169***	− 0.035	− 0.219***	0.025
	(0.065)	(0.070)	(0.067)	(0.071)
HighCost × Arguments (n = 97)	0.192***	0.131*	− 0.188***	− 0.149**
	(0.067)	(0.069)	(0.068)	(0.070)
HighCost × Facts (n = 100)	0.151**	0.063	− 0.168**	− 0.051
	(0.065)	(0.070)	(0.067)	(0.070)
HighCost × Arguments + Facts (n = 100)	0.113*	0.051	− 0.161**	− 0.067
	(0.065)	(0.071)	(0.067)	(0.071)
Mean of LowCost × NoInfo (n = 102)	0.265***	0.461***	0.706***	0.510***
	(0.044)	(0.049)	(0.045)	(0.050)
Total observations (N)	803	803	803	803

TEP refers to pricing strategy that is tiered across countries, equitable for poorer countries, and profitable for manufacturers. Conflicted refers to conflicted participants who supported some but not all the three components of TEP (tiering, equity, and profitability). Personal decisions are decisions made in the individual task. Coordination decisions are decisions made in the coordination (collective agreement) task. The coefficient estimate for each treatment group is the mean difference between the share of a decision in the treatment group and the share of a decision in the baseline LowCost × NoInfo group. All estimates are based on a multivariate Ordinary Least Squares regression that include all treatment indicators, dummies for sufficient baseline knowledge of factors relevant to vaccine pricing, total quiz score (which measures comprehension of experimental instructions), and an attention check indicator (which controls for whether participants paid attention during the experiment). The means for the baseline LowCost × NoInfo group are reported in the row above the number of observations (N). The sample size in each group is denoted by n. Robust standard errors are reported in parentheses. *** *p* < 0.01; ** *p* < 0.05; * *p* < 0.10.

### Difference between individual-task and coordination-task

The incentive to arrive at collective agreement in the coordination task might have by itself motivated participants to support both equity and profitability considerations, regardless of vaccine cost and explicit provision of information about equity and profitability considerations. Such motivation might be muted in the individual task and sensitive to vaccine cost, which in turn might make participants feel conflicted about supporting both equity and profitability as embodied in TEP. To examine this possibility, we defined a participant as “conflicted” if they supported some but not all three underlying components of TEP (i.e., tiering, equity, and profitability).

Column 3 in Table 2 reports the change in the share of conflicted participants in any treatment relative to the share of conflicted participants in the LowCost × NoInfo baseline for the individual task. An increase in vaccine cost or provision of information in all cases significantly decreased the share of conflicted participants compared to the LowCost × NoInfo baseline in the individual task. Thus, information provision reduced the perceived conflict in supporting both equity and profitability and increased the support for TEP of low-cost vaccines from the personal perspective. However, in the coordination task (column 4 in Table 2), except in the HighCost × Arguments treatment where the effect was negative (−14.9 percentage points; *p* = 0.033), the cost and information treatments had no effect on the share of conflicted participants relative to the LowCost × NoInfo baseline. Overall, these results are consistent with the interpretation that the incentive to arrive at collective agreement in the coordination task sufficiently motivates participants to support both equity and profitability considerations.

## Discussion

Our survey experiment with a demographically representative sample of US adults demonstrated that TEP was the personally most preferred global vaccine pricing strategy out of all pricing strategies. Without information about the importance of both equity across countries and profitability for manufacturers in global vaccine pricing, personal support for TEP of low-cost-vaccines was relatively low. Providing information significantly increased personal support for TEP of low-cost vaccines by reducing the perceived conflict in supporting both equity and profitability considerations in vaccine pricing. Information campaigns can thus be helpful in increasing personal support for TEP of vaccines at later stages in the product cycle when vaccine prices tend to be lower. Similarly, information campaigns can also be helpful in increasing personal support for TEP of low-cost vaccines for a wide-range of vaccine-preventable diseases, such as tuberculous meningitis and measles, that disproportionately burden poorer countries.

Our study also revealed that TEP was the pricing strategy most likely to achieve collective agreement, regardless of vaccine cost and provision of information. This suggests that the need to meet both equity and profitability considerations in global vaccine pricing becomes salient when the goal is to identify a collectively agreeable global vaccine pricing strategy, regardless of vaccine cost and provision of information. One implication of these findings relates to the framing of public discourse regarding global vaccine pricing. Framing these debates as an issue that calls for collective agreement may enhance support for TEP, especially in the context of infectious diseases where it may be easier to convey the message that no one is safe until everyone is safe.

Our study has several limitations. First, the study used an online survey experimental methodology. Although this approach facilitated the timely implementation of the survey experiment on a demographically representative sample of the US adult population, participants were relatively skilled in using computers or smart phones, and had access to the internet. The findings may not be generalizable to population subgroups that do not share these characteristics. Similarly, whether our findings hold in other rich countries remains an open question for future research. Second, as the experiment involved only two cost conditions and four information conditions, the findings may not be applicable in other possible cost and information conditions. Third, our study is underpowered for most treatment conditions at *α* = 0.05 and *β* = 0.8. The minimum sample size that is required for the detection of statistical treatment differences is larger than 100 for most comparisons. It is possible the standard errors of the estimates may become smaller with larger sample sizes. Further research with larger sample sizes is needed to assess the robustness of our findings. Fourth, asking the participants questions about their knowledge of relevant facts in the pre-experiment survey may have affected their support for TEP. While we did not provide any feedback to the participants, the potential impact of even asking such questions on the support for TEP cannot be completely ruled out. Lastly, we must acknowledge that public support for TEP in high-income countries is unlikely to have any immediate and meaningful impact on the mechanisms of GAVI or COVAX.

Despite these limitations, it is worth noting that tiered pricing that is profitable for manufacturers but not equitable for poorer countries (TPnotE) has long been criticized by public health and health policy experts as a barrier to global vaccine equity^10–13^. Our finding that there is greater public support for TEP relative to TPnotE suggests that people in the US broadly agree with this criticism too. Our findings also suggest that it can be useful to employ information campaigns to improve awareness of equity and profitability considerations in global vaccine pricing and to frame public discourse about TEP as an issue that calls for collective agreement on a global vaccine pricing strategy.

## Supplementary Information

Supplementary Information.

## Data Availability

The datasets generated during and/or analysed during the current study are available from the corresponding author upon request.
