# Supplementary material for: Public support in the United States for global equity in vaccine pricing
Source: Sci Rep. 2022 May 27;12:8960. doi: 10.1038/s41598-022-13172-7 (PMC9137445; doi:10.1038/s41598-022-13172-7)
Supplement: Supplementary file 1 — Supplementary Information. [file 41598_2022_13172_MOESM1_ESM.docx]

**Supplementary information**

Section 1. Materials and methods

Section 2. Analysis

Table S1. Outline of the experiment

Table S2. Summary statistics and balance test

Table S3. Selected quiz scores

Instructions to participants

References

# Materials and methods

## Experimental methodology

Global vaccine pricing is contentious because of the need to balance affordability for poorer countries and profitability for manufacturers. Although tiered pricing that is equitable across countries and profitable for manufacturers (TEP) can promote global vaccine equity, there are barriers to implementation, including the potential lack of public support in richer countries.

We searched PubMed and Google Scholar on November 15, 2020 using the search terms “vaccine price” AND “survey*” OR “experiment*” OR “information”. We searched for original research and reviews published in English from database inception up to the date of the literature search. We also conducted Google search using these keywords. The search was repeated on September 15, 2021.

We found a few studies on the price of vaccines in some high-income countries. These were surveys and survey experiments designed to estimate the willingness to pay (WTP) for vaccines in a range of countries, and to identify the main factors affecting the WTP or demand for vaccination more generally. Estimates on the mean WTP varied by country and infectious disease. The WTP ranged from 46 euros (approximately 54 USD) for vaccines against Tick-Borne Encephalitis in Sweden^1^ to 100-200 US dollars for the COVID-19 vaccine in the US, UK, Spain and Italy,^2^ and more than USD 230 in Chile.^3^ These studies indicate the WTP of people in high-income countries, but do not inform us about their views on global vaccine pricing. To the best of our knowledge, no prior study has examined the extent and determinants of public support in high income countries for TEP.

Table S1 summarises the structure of the experiment. Participants were randomly assigned to one of eight treatments that varied the vaccine cost (low or high) and four types of information relating to equity and profitability considerations in global vaccine pricing (argument-based, fact-based, fact-and-argument-based, and no information). We provide more detail about each component of the experiment below.

**Pre-experiment survey**. Participants answered questions about their experience with Covid-19, attitude towards vaccination, views on income equality and global poverty, and familiarity with vaccine pricing. The question about familiarity with vaccine pricing indicates that 48% of participants had never heard of factors relevant to the pricing of vaccines. Participants were then asked six multiple-choice questions about low and lower-middle income countries (knowledge quiz), earning a bonus payment of GBP 0.10 for each correct answer. We asked participants to guess: (i) the average income per day in low and lower-middle income countries, (ii) share of the world’s population in low and lower-middle income countries, (iii) share of world’s extremely poor people in low and lower-middle income countries, (iv) share of the world’s vaccine-preventable deaths from infectious diseases (excluding Covid-19) in low and lower-middle income countries, (v) eligibility status of low-income countries for GAVI support to access vaccines, (vi) eligibility status of lower-middle income countries for GAVI support to access vaccines. The multiple-choice options in each question were randomized. The average knowledge quiz score was 1.91 out of 6, which suggests that participants had a low level of prior knowledge about GAVI and factors relevant for TEP and GAVI before the experiment. When we combine participants’ positive response to the question about familiarity with vaccine pricing and their baseline knowledge quiz score, we find only 17% of them score at least four out of seven on this composite measure which we refer to as the measure of “sufficient baseline knowledge”. The evidence suggests “sufficient baseline knowledge” is low and balanced across all treatment groups.

**Explanation and quiz**. We explained to participants the setting of the experiment, and the type of concepts and situations that they would encounter during the choice experiment. The explanation was presented along with a series of incentivized comprehension questions. Participants earned a bonus of GBP 0.10 per correct answer, which provided incentives to carefully read and understand the structure of the situations in the actual experiment. The correct answer to a quiz question was provided immediately after a participant answered a quiz question, regardless of whether the participant had answered correctly or incorrectly. We also re-tested their understanding in three additional comprehension questions. As Table S3 shows, the average number of correct responses was 2.6 out of 3, which suggests that participants had a clear understanding of the basic concepts and setting of the choice experiment.

**Arguments**. Participants who were allocated to the argument-based treatment groups were provided with arguments that highlighted the tension between equity and profitability considerations in global vaccine pricing. Specifically, the arguments were that prices should be based on a country’s need and ability to pay, and provide incentives for pharmaceutical firms to undertake vaccine R&D. These arguments were drawn from the actual pricing policies of some of the largest vaccine manufacturers in the U.S.^4-8^ After presenting each argument, we asked participants to what extent they agree with a statement about the argument using a 5-point Likert scale. This was done to encourage participants to read the information and think about the arguments. All three arguments were framed using neutral language, presented on the same page but in a random order across participants.

Participants in the arguments-based group were provided with arguments containing no reference to facts, and read the following:

**Factor:** The overall pricing strategy of vaccines across different countries should aim to make the vaccine affordable for all countries and be based on a country's **ability to pay**.

It has been argued that vaccine pricing should ensure that low-income countries pay the lowest price.

How strongly do you agree or disagree with the following statement?

"High-income countries should pay the most for vaccines."

o Strongly disagree

o Disagree

o Neither agree nor disagree

o Agree

o Strongly agree

**Factor:** The overall pricing strategy of vaccines across different countries should aim to make vaccines available to countries that **need** them the most as quickly as possible.

It has been argued that vaccine pricing should ensure that lower middle-income countries have adequate access to vaccines.

How strongly do you agree or disagree with the following statement?

"Lower middle-income countries should receive adequate support to access vaccines, and pay a price for vaccines that allows them to prevent deaths and reduce the burden of extreme poverty."

o Strongly disagree

o Disagree

o Neither agree nor disagree

o Agree

o Strongly agree

**Factor:** Firms should have sufficient **incentive to continue to research, develop, innovate and supply** vaccines.

It has been argued that the combination of government support and vaccine pricing should ensure that firms are appropriately encouraged and rewarded for investment in vaccine research and development.

How strongly do you agree or disagree with the following statement?

"The combination of government support and vaccine pricing should give firms sufficient incentive to continue to research, develop, innovate and supply vaccines to meet global health needs."

o Strongly disagree

o Disagree

o Neither agree nor disagree

o Agree

o Strongly agree

**Facts**. Participants who were allocated to the fact-based and fact-and-argument based treatment groups were provided with facts that may be relevant to the consideration of equity and profitability in global vaccine pricing. The facts were a series of statistics shown in three tables.

We presented two tables of statistics relevant to the affordability of vaccines. The first table showed data from the World Bank on the average income per day for each of the 4 income-based country types.^9^ The second table showed data on: (i) the share of the world’s population by country type,^10^ (ii) the share of the world’s extremely poor people by country type,^11^ (iii) the share of the world’s vaccine-preventable deaths from several infectious diseases (excluding COVID-19) by country type,^12^ (iv) eligibility for GAVI assistance to access vaccines by country type.^13^ The 14 infectious diseases were selected based on the list of diseases for which vaccines are currently available and published by the WHO, and for which data were available.^14^ The diseases were: Tuberculosis, Whooping cough, Tetanus, Measles, Varicella and herpes zoster, Acute hepatitis A, Acute hepatitis B, Typhoid fever, Pneumococcal meningitis, H influenzae type B meningitis, Meningococcal meningitis, Diphtheria, Dengue, and Yellow fever. We chose to exclude COVID-19 because the infectious disease was still running its course at the time of the experiment, was not vaccine preventable for most of 2020, and the global supply of vaccines remained relatively limited.

The last table of statistics was related to the profitability of vaccines. This table showed data for vaccines, cardiovascular disease drugs, and cancer drugs on: (i) the average cost of R&D,^15–17^ (ii) the average time for development,^18–20^ (iii) the probability of success from development to regulatory approval,^18,21,22^ (iv) global pharmaceutical market value.^23–25^ We chose these pharmaceutical products as they are comparable to vaccines along the key R&D characteristics.

After presenting each table, we asked participants a comprehension question about the statistics to check that they had read and understood the data. As Table S3 shows, comprehension was relatively high, with the average score being 2.8 out of 3. The facts were presented in random order on the same page. Participants read the following:

According to the World Bank, the average income of people across all countries around the world is as follows:

| Country | Average income per person per day in 2019 (USD) |
| --- | --- |
| Low income | 2 |
| Lower middle-income | 6 |
| Upper middle-income | 24 |
| High income | 124 |

Based on the above description, which of the following statements is correct?

o Low-income countries have an average income of USD 2 per person per day.

o Lower middle-income countries have an average income of USD 2 per person per day.

o Upper middle-income countries have an average income of USD 2 per person per day.

o High-income countries have an average income of USD 2 per person per day.

According to the World Bank and Institute for Health Metrics and Evaluation, the distribution of the world's population, extremely poor people, and vaccine-preventable deaths is given by the table below.

| Country | % world’s  population in 2019 | % world’s  extremely poor in 2019* | % world’s  vaccine-preventable deaths in 2017** | Eligibility for  GAVI support to access vaccines*** |
| --- | --- | --- | --- | --- |
| Low income | 9 | 36 | 24 | Yes |
| Lower middle-income | 38 | 58 | 67 | No |
| Upper middle-income | 37 | 5 | 8 | No |
| High income | 16 | 1 | 1 | No |

*Note: people living on less than USD 1.90 per day

**Note: several infectious diseases excluding COVID-19

***Note: support includes vaccines in-kind and financial support

GAVI is an international organization that aims to improve access to vaccines for children living in the world's poorest countries. Currently, only low-income countries are eligible for GAVI support to access vaccines, although they are still required to contribute to the cost of vaccines.

Based on the above description, which of the following statements is correct?

o Low-income countries have the majority of the world's extremely poor people and vaccine-preventable deaths, and are not eligible for GAVI support to access vaccines.

o Lower middle-income countries have the majority of the world's extremely poor people and vaccine-preventable deaths, and are not eligible for GAVI support to access vaccines.

o Upper middle-income countries have the majority of the world's extremely poor people and vaccine-preventable deaths, and are not eligible for GAVI support to access vaccines.

o High-income countries have the majority of the world's extremely poor people and vaccine-preventable deaths, and are not eligible for GAVI support to access vaccines.

According to estimates from the World Health Organization, researchers and industry, some characteristics of the development and global market value of selected pharmaceutical products are as follows:

| Pharmaceutical product | Average cost of research & development (USD) | Average time for development (years) | Probability of success from development to regulatory approval (%) | Global pharmaceutical market value in 2018 (USD) |
| --- | --- | --- | --- | --- |
| Vaccines* | 300-500 million | 11 | 6 | 26 billion |
| Cardiovascular disease drugs | 1 billion | 12 | 7 | 47 billion |
| Cancer drugs | 5 billion | 7** | 3 | 124 billion |

*Note: several infectious diseases excluding COVID-19

**Note: median

Based on the above description, which of the following statements is correct?

o Cardiovascular disease drugs had the lowest global pharmaceutical market value in 2018.

o Cancer drugs had the lowest global pharmaceutical market value in 2018

o Vaccines had the lowest global pharmaceutical market value in 2018.

**Facts and Arguments**. Participants in the facts-and-arguments group were presented with the same arguments as those in the arguments group, except that the arguments now referred to supporting facts and provided reasons to support the argument. They read the following:

**Factor:** The overall pricing strategy of vaccines across different countries should aim to make the vaccine affordable for all countries and be based on a country's **ability to pay**.

It has been argued that vaccine pricing should ensure that low-income countries pay the lowest price because they have the lowest average income.

How strongly do you agree or disagree with the following statement?

"High-income countries should pay the most for vaccines."

o Strongly disagree

o Disagree

o Neither agree nor disagree

o Agree

o Strongly agree

**Factor:** The overall pricing strategy of vaccines across different countries should aim to make vaccines available to countries that **need** them the most as quickly as possible.

It has been argued that vaccine pricing should ensure that lower middle-income countries have adequate access to vaccines because they are not eligible for GAVI support to access vaccines, despite having the majority of the world's extremely poor people and vaccine-preventable deaths.

How strongly do you agree or disagree with the following statement?

"Lower middle-income countries should receive adequate support to access vaccines, and pay a price for vaccines that allows them to prevent deaths and reduce the burden of extreme poverty."

o Strongly disagree

o Disagree

o Neither agree nor disagree

o Agree

o Strongly agree

**Factor:** Firms should have sufficient **incentive to continue to research, develop, innovate and supply** vaccines.

It has been argued that the combination of government support and vaccine pricing should ensure that firms are appropriately encouraged and rewarded for costly and risky investment in vaccine research and development because it usually takes many years and has a high failure rate, but has low global market value.

How strongly do you agree or disagree with the following statement?

"The combination of government support and vaccine pricing should give firms sufficient incentive to continue to research, develop, innovate and supply vaccines to meet global health needs."

o Strongly disagree

o Disagree

o Neither agree nor disagree

o Agree

o Strongly agree

**Personal decisions**. We asked participants about their personal views on vaccine pricing and firm’s profits across different countries. All participants were presented with a hypothetical scenario of a global pandemic which had two key assumptions: (i) a firm had developed a safe and effective vaccine that had been approved by health authorities, (ii) the firm had the ability to supply the whole world. Participants were also told about the firm’s cost of producing the vaccine (low or high), which differed by treatment group. They were given three pieces of information about costs: (i) total cost per dose, (ii) production (marginal) cost per dose, (iii) pre-production (fixed) cost per dose.

Participants were asked to choose the price per dose of vaccine that the firm should charge low-income, lower-middle income, upper-middle income and high-income countries based on the World Bank’s income classification.^26^ For each country type, participants could choose among the following six price categories (from lowest to highest): (i) below marginal cost, (ii) equal to marginal cost, (iii) above marginal cost but significantly less than total cost, (iv) significantly above marginal cost but less than total cost, (v) equal to total cost, (vi) above total cost. We used the midpoint between the marginal cost and total cost to delineate between price category (iii) and price category (iv). The profit implication of choosing a particular price category was also noted alongside each category. For example, participants were told that choosing price category (vi) for a country would imply the firm would make profit from that country. In the low-cost treatment, participants read the following when asked about what price to charge for a low-income country:

The expected **total cost** per dose is **USD 10**. The breakdown of the expected total cost per dose is as follows:

- Expected production cost per dose = **USD 3**
- Pre-production cost per dose = **USD 7**

In your view, what price per dose should the firm charge a **low-income country**?

Please select one of the following options.

o **Less than USD 3.** The firm makes an overall loss per dose.

o **Equal to USD 3.** The firm makes an overall loss per dose.

o **More than USD 3 but less than USD 6.50.** The firm makes an overall loss per dose.

o **More than USD 6.50 but less than USD 10.** The firm makes an overall loss per dose.

o **Equal to USD 10.** The firm breaks even and makes neither an overall profit nor overall loss per dose.

o **More than USD 10.** The firm makes some overall profit per dose.

In the high-cost treatment, participants were asked the same questions, except the cost figures were replaced with higher numbers (total cost per dose of USD 50, expected production cost per dose of 15, and pre-production cost per dose of 35).

Finally, participants were asked whether the firm should make some “overall profit” from its pricing across all countries. They could choose one out of three options: Yes, No, or Unsure. For example, in the low-cost treatment, participants read the following:

In this scenario, the expected **total cost** per dose is **USD 10**. The breakdown of the expected total cost per dose is as follows:

- Expected production cost per dose = **USD 3**
- Pre-production cost per dose = **USD 7**

In your view, should the firm make some overall profit from its pricing across different countries?

Please select one of the following options.

o Yes, the firm should make at least some overall profit

o Unsure

o No, the firm should make no overall profit

In total, participants answered five questions (four pricing questions for each country type and one question on profits). We used this format to elicit preferences for vaccine prices and profitability instead of asking participants to report exact prices for two reasons. First, we wished to encourage participants to focus on the tension between equity and profitability rather than on precise numerical calculations. Second, we wanted to avoid any potential calculation errors by participants.

**Coordination decisions**. In this stage, participants were presented with the same hypothetical situation and same questions they encountered in the personal preference stage. However, they were told that they would earn a bonus of GBP 0.10 for each question if their choice of prices and profits matched the most frequent choice of all participants in a given cost scenario.

The payment structure of the coordination stage provided participants with incentives to think about which prices and profits were most likely to be chosen by other participants in the experiment. Therefore, the participants’ decisions in this stage not only allow us to understand what participants believed other participants would choose, but also infer which price or profit is socially focal (most likely to be agreed upon by all participants), which may be different from an individual’s personal preference.

Before participants could proceed to the coordination game, they had to answer a quiz question about how they would be paid in this stage. This served as an attention-check, and also helped assess whether participants understood the incentive mechanism used to determine their payment in the coordination games. Only 6.4% of the participants (51 out of 803) answered this question incorrectly. The findings are robust to controlling for responses to this attention-check question.

**Post-experiment survey**. The final component of the experiment asked participants questions about their demographic, socioeconomic, and attitudinal characteristics such as age, gender, educational attainment, and political leaning. Participants also responded to five questions commonly used in the Cognitive Reflection Test (CRT), and earned a bonus of GBP 0.10 for each correct answer.

## Subject pool

The randomized controlled choice experiment was conducted online in the US between April 17, 2021 and May 21, 2021 via Prolific, a UK research survey platform. The experiment was programmed using Qualtrics. Prolific has approximately 36,000 US participants available for research studies. At the time that we conducted the experiment, there were approximately 24,000 eligible participants in the US who were deemed active (had participated in a study in the past 90 days) and provided sufficient information to Prolific about their characteristics for us to generate a demographically representative sample.

Once a research study is published on Prolific, Prolific makes them visible to the eligible participants and sends out emails to those who subscribe for notifications. Participants interested in our study would then need to log into Prolific, select our study, and launch the survey. A total of 844 respondents launched the survey, of which 41 (4.9%) did not finish the survey, including those who quit the survey at the consent page. Of those who stayed in the survey long enough to be assigned a treatment status, the attrition rate was 1.4% (11 out of 814 respondents). While these 11 respondents answered more than the quiz questions and the explanation, only 1 respondent moved beyond the personal choice decisions. Overall, attrition rates were broadly similar between the “LowCost×NoInfo” baseline group and 7 treatment groups (1 respondent in each treatment group, except for 4 respondents in the “HighCost×NoInfo” treatment group). Moreover, when respondents began the survey, they did not know which treatment group they had been assigned to.

Our final sample consists of the 803 participants who finished the survey. There were approximately 100 participants in each of the 8 treatment groups. Each participant was paid GBP 2.10 for participation, and could earn up to an additional GBP 2.90 depending on their coordination decisions, and performance on the quiz and CRT.

## Key design choices

We made two key choices in the experimental design: the cost of producing vaccines and definition of equitable tiered pricing. As we already explained the latter in the main text, we will now explain our choices of vaccine costs (USD 10 and USD 50 per dose).

We chose to have two cost scenarios (low and high) as there is significant variation in the price of vaccines for several infectious diseases and over time,^27^ which in part reflects differences in costs. The specific cost levels that we chose (USD 10 and USD 50) are based on price data reported by the WHO,^28^ as we found the highest price paid by poorer countries was about USD 10 per dose, while the highest price paid by richer countries was about USD 50 per dose. In both scenarios, the production (marginal) cost was set at 30%, while the pre-production (fixed) cost was set at 70% to reflect the typically high costs of vaccine R&D.^15,16,29,30^

# Analysis

## 2.1 Descriptive statistics

Table S2 reports the summary statistics for our sample compared to the 2019 American Community Survey (ACS) for the US population aged 18 or over.^31^ Our sample is broadly similar to the US adult population in terms of age (above or below 47 years), gender (male or female) and race (white or non-white). However, respondents in our sample are more likely to have a college degree or higher than the general population.

We also checked for balance by comparing the mean characteristics of the “LowCost×NoInfo” baseline group and the 7 treatment groups, and testing for differences using t-tests and F-tests of joint significance. Given the random assignment of the cost and information treatments, there is no statistically significant difference between the “LowCost×NoInfo” baseline group and each of the 7 treatment groups with respect to their observable characteristics. These characteristics include: gender, race, age, education, knowledge quiz score, familiarity with vaccine pricing, vaccine hesitancy, political orientation, background quiz score, attention check score, and CRT score.

## 2.2 Regression

To explore the effects of the cost-and-information treatments on support for TEP and alternative pricing strategies, we compared the decisions by participants in each of the seven treatment groups with the “LowCost×NoInfo” baseline group by estimating the following equation using multivariate ordinary least squares (OLS) regressions:

$$y_{i}=\alpha+\beta_{1}{LowCostArgs}_{i}+\beta_{2}{LowCostFacts}_{i}+\beta_{3}{LowCostBoth}_{i}+\beta_{4}{HighCostNoInfo}_{i}+\beta_{5}{HighCostArgs}_{i}+\beta_{6}{HighCostFacts}_{i}+\beta_{7}{HighCostBoth}_{i}+\theta knowledge_{i}+\delta{quizscore}_{i}+\gamma{attention}_{i}+\varepsilon_{i} (1)$$

We included all 7 treatment groups (omitting the “LowCost×NoInfo” baseline group) as binary indicator variables that equal 1 if respondent *i* was assigned to the relevant cost-and-information treatment group, and 0 otherwise. Given that the quality of responses is likely to be affected by a participant’s understanding of the experimental instructions and attention during the experiment, as well as to minimize noise, the estimation includes controls for whether a participant has sufficient baseline knowledge about factors relevant to vaccine pricing, participant background quiz scores, and a binary indicator for whether a participant paid attention during the experiment. We estimated equation (1) separately for the personal choice experiment and the coordination game, and used robust standard errors clustered at the participant level.

The dependent variable $y_{i}$ is a binary indicator variable measuring the outcome of interest. At the individual level, the main outcome is support for TEP (Column 1 in Table 2). The corresponding support for TEP in coordination decisions are reported in Column 2 of Table 2. To better understand the mechanism behind the results, we examined the effect of the cost-and-information treatments on decisions that indicate support for some but not all three components of TEP (tiering, equity, and profitability). We coded participants as “conflicted” if they supported one or two but not all three components of TEP. The results are reported for both personal decisions and coordination decisions in Columns 3 and 4 of Table 2 respectively.

The constant $\alpha$ in the OLS regression measures the mean share of participants supporting the outcome of interest in the “LowCost×NoInfo” baseline group. The coefficients of interest are $\beta_{1}$ to $\beta_{7}$, which measure the average effect of the cost-and-information treatments on the probability of participants supporting the outcome of interest relative to the “LowCost×NoInfo” baseline group.

Table S1. Outline of the experiment

|  | LowCost×  NoInfo (baseline) | LowCost×  Arguments | LowCost×  Facts | LowCost×  Arguments+Facts | HighCost×  NoInfo | HighCost×  Arguments | HighCost×  Facts | HighCost×  Arguments+Facts |
| --- | --- | --- | --- | --- | --- | --- | --- | --- |
| Pre-experiment survey | Yes | Yes | Yes | Yes | Yes | Yes | Yes | Yes |
| Explanation and quiz | Yes | Yes | Yes | Yes | Yes | Yes | Yes | Yes |
| Arguments | No | Yes | No | Yes | No | Yes | No | Yes |
| Facts | No | No | Yes | Yes | No | No | Yes | Yes |
| Personal decisions | Yes | Yes | Yes | Yes | Yes | Yes | Yes | Yes |
| Coordination decisions | Yes | Yes | Yes | Yes | Yes | Yes | Yes | Yes |
| Post-experiment survey | Yes | Yes | Yes | Yes | Yes | Yes | Yes | Yes |

Notes: The baseline is the “LowCost×NoInfo” group.

**Table S2. Summary statistics and balance test**

| Characteristics | ACS 2019 | LowCost×  NoInfo (baseline) | LowCost×  Arguments | LowCost×  Facts | LowCost×  Arguments+  Facts | HighCost×  NoInfo | HighCost×  Arguments | HighCost×  Facts | HighCost×  Arguments+  Facts | F-stat |
| --- | --- | --- | --- | --- | --- | --- | --- | --- | --- | --- |
| Male | 0.487 | 0.451 | 0.485 | 0.545 | 0.461 | 0.450 | 0.454 | 0.550 | 0.500 | 0.68 |
|  |  | (0.050) | (0.050) | (0.050) | (0.050) | (0.050) | (0.051) | (0.050) | (0.050) |  |
| White | 0.736 | 0.745 | 0.743 | 0.663 | 0.686 | 0.820 | 0.732 | 0.740 | 0.750 | 1.20 |
|  |  | (0.043) | (0.044) | (0.047) | (0.046) | (0.039) | (0.045) | (0.044) | (0.044) |  |
| Age over 47 years | 0.491 | 0.490 | 0.436 | 0.416 | 0.520 | 0.530 | 0.557 | 0.490 | 0.490 | 0.88 |
|  |  | (0.050) | (0.050) | (0.049) | (0.050) | (0.050) | (0.051) | (0.050) | (0.050) |  |
| College degree or higher | 0.389 | 0.529 | 0.495 | 0.564 | 0.490 | 0.570 | 0.515 | 0.530 | 0.520 | 0.34 |
|  |  | (0.050) | (0.050) | (0.050) | (0.050) | (0.050) | (0.051) | (0.050) | (0.050) |  |
| Heard of  vaccine pricing |  | 0.549 | 0.515 | 0.485 | 0.500 | 0.530 | 0.515 | 0.510 | 0.570 | 0.30 |
|  |  | (0.050) | (0.050) | (0.050) | (0.050) | (0.050) | (0.051) | (0.050) | (0.050) |  |
| Baseline knowledge of factors relevant for TEP |  | 1.922 | 2.030 | 1.891 | 1.892 | 1.940 | 1.763 | 1.960 | 1.880 | 0.59 |
|  |  | (0.096) | (0.108) | (0.097) | (0.104) | (0.101) | (0.095) | (0.106) | (0.099) |  |
| Sufficient baseline knowledge |  | 0.196 | 0.218 | 0.149 | 0.157 | 0.170 | 0.113 | 0.200 | 0.180 | 0.85 |
|  |  | (0.040) | (0.041) | (0.036) | (0.036) | (0.038) | (0.032) | (0.040) | (0.039) |  |
| Vaccine hesitant |  | 0.216 | 0.188 | 0.198 | 0.245 | 0.190 | 0.237 | 0.290 | 0.170 | 0.84 |
|  |  | (0.041) | (0.039) | (0.040) | (0.043) | (0.039) | (0.043) | (0.046) | (0.038) |  |
| COVID-19 experience |  | 1.324 | 1.356 | 1.257 | 1.363 | 1.400 | 1.361 | 1.200 | 1.210 | 0.75 |
|  |  | (0.094) | (0.089) | (0.095) | (0.081) | (0.089) | (0.087) | (0.089) | (0.087) |  |
| Republican-leaning |  | 0.255 | 0.297 | 0.337 | 0.294 | 0.280 | 0.320 | 0.280 | 0.280 | 0.31 |
|  |  | (0.043) | (0.046) | (0.047) | (0.045) | (0.045) | (0.048) | (0.045) | (0.045) |  |
| Background quiz score |  | 11.147 | 10.792 | 11.267 | 11.147 | 11.170 | 10.742 | 10.880 | 11.460 | 1.04 |
|  |  | (0.227) | (0.261) | (0.194) | (0.265) | (0.252) | (0.291) | (0.267) | (0.212) |  |
| Passed attention check |  | 0.951 | 0.941 | 0.921 | 0.941 | 0.980 | 0.948 | 0.920 | 0.890 | 1.55 |
|  |  | (0.021) | (0.024) | (0.027) | (0.023) | (0.014) | (0.023) | (0.027) | (0.031) |  |
|  |  |  |  |  |  |  |  |  |  |  |
| High CRT score |  | 0.510 | 0.386* | 0.525 | 0.461 | 0.490 | 0.381 | 0.440 | 0.490 | 1.20 |
|  |  | (0.050) | (0.049) | (0.050) | (0.050) | (0.050) | (0.050) | (0.050) | (0.050) |  |
| Observations | 2,599,171 | 102 | 101 | 101 | 102 | 100 | 97 | 100 | 100 |  |

Notes: Participant characteristics are reported as means and in fractions. The baseline is the “LowCost×NoInfo” group. American Community Survey (ACS) 2019 data are based on the US population aged 18 years or over.^31^ Standard errors of the means clustered at the participant level are reported in parentheses. ***, **, and * indicate a t-test on the difference in means between the baseline and relevant treatment group is significant at the 1, 5 and 10% level respectively. F-statistic is from a F-test of joint significance from regressing each characteristic on a constant and 7 treatment groups, and testing whether the treatment groups jointly explain the characteristic.

Table S3. Selected quiz scores

|  | LowCost×  NoInfo (baseline) | LowCost×  Arguments | LowCost×  Facts | LowCost×  Arguments+Facts | HighCost×  NoInfo | HighCost×  Arguments | HighCost×  Facts | HighCost×  Arguments+Facts |
| --- | --- | --- | --- | --- | --- | --- | --- | --- |
| Mean score on 3 extra questions in background quiz (re-testing) | 2.569 | 2.545 | 2.624 | 2.618 | 2.510 | 2.536 | 2.480 | 2.670 |
| Mean score on 3 comprehension questions in “facts” treatment |  |  | 2.812 | 2.843 |  |  | 2.820 | 2.790 |
| Mean time spent on information treatments (minutes) |  | 1.412 | 2.631 | 4.679 |  | 1.387 | 2.735 | 4.104 |

Notes: The baseline is the “LowCost×NoInfo” group.

**Instructions to participants**

**Start of Block: Introduction (Prolific)**

Welcome and thank you for your participation in this research study.

You will be paid GBP 2.10 for completing this study, and you may earn up to an additional GBP 2.90 depending on your responses. Thus, you can earn a total of at least GBP 2.10 and at most GBP 5.00 from completing this study.

- You must attempt this survey **only once**. If you attempt this survey more than once, then you will not be eligible for payment.
- Once you begin this survey, you will have **75 minutes to complete** it. If you do not finish the survey within 75 minutes after starting the survey, then you will not be eligible for payment.

The purpose of this study is to understand the views of people regarding the global allocation and pricing of vaccines during a pandemic.

It contains several questions, some of which may check that you are paying attention.

The survey will take you approximately 25 minutes to finish.

Your payment will be processed by the researchers within 5 days from the time this study is over.

Before starting, please read the following Consent Form.

**Consent Form**

Do you agree to the above terms?

If you do not wish to participate, please close this window.

By clicking YES, you consent that you are willing to answer the questions in this survey.

- Yes, I agree

Please confirm your Prolific ID Number, and then click next to proceed.

- You must attempt this survey **only once**.
- If you attempt this survey more than once, then you will not be eligible for payment.

________________________________________________________________

**End of Block: Introduction (Prolific)**

**Start of Block: Pre-Survey Questions**

We will ask you some questions about yourself.

How would you place your views on this scale?

Click or drag the slider to indicate your views. 1 means you agree completely with the statement on the left; 10 means you agree completely with the statement on the right; and if your views fall in between, then choose any number in between accordingly.


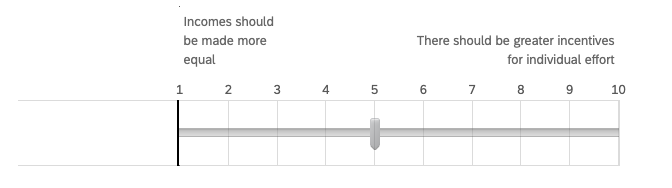


How would you place your views on this scale?

Click or drag the slider to indicate your views. 1 means you agree completely with the statement on the left; 10 means you agree completely with the statement on the right; and if your views fall in between, then choose any number in between accordingly.


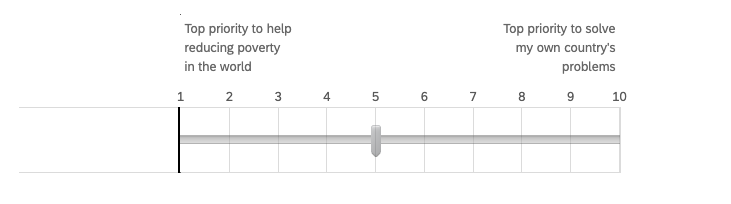


Have you been vaccinated with a COVID-19 vaccine (at least 1 dose)?

- Yes
- No

Suppose there is a COVID-19 vaccine that is considered to be safe and effective by health authorities. When you are eligible to receive a vaccine, would you take this vaccine if the government offers it to you for free?

- Yes
- Unsure
- No

Did you personally have COVID-19 at any point?

- Yes, and I was hospitalised
- Yes, but I didn't require hospitalisation
- No

Do you personally know anyone who has had COVID-19?

- Yes
- No

Do you personally know anyone who has been hospitalised or died due to COVID-19?

- Yes
- No

How familiar are you with the factors relevant to the pricing of vaccines?

- Never heard of them before
- Heard of the factors, but don't know the details
- Somewhat familiar with the details
- Very familiar with the details

**End of Block: Pre-Survey Questions**

**Start of Block: Pre-Survey Knowledge Quiz Preamble**

We will now describe some factors and ask you some questions that may be relevant to the price of vaccines across different countries.

In **some** questions, you will earn a **bonus of GBP 0.10** if your answer is correct.

**End of Block: Pre-Survey Knowledge Quiz Preamble**

**Start of Block: Pre-Survey Knowledge Quiz**

International agencies such as the World Bank classify countries around the world into 4 groups based on the average income of people in a country:

- Low-income countries have the lowest average income per person
- Lower middle-income countries have the second-lowest average income per person
- Upper middle-income countries have the second-highest average income per person
- High-income countries have the highest average income per person.

Based on the above description, which of the following statements is correct?

You will earn a **bonus of GBP 0.10** if your answer is correct.

- High-income countries have the lowest average income per person.
- Upper middle-income countries have the highest average income per person.
- Low-income countries have the lowest average income per person.
- Lower middle-income countries have the lowest average income per person.

Your answer is **CORRECT**. You have just earned an additional GBP 0.10.

Low-income countries have the lowest average income per person.

Lower middle-income countries have the second-lowest average income per person.

Upper middle-income countries have the second-highest average income per person.

High-income countries have the highest average income per person.

Your answer is **INCORRECT**.

Low-income countries have the lowest average income per person.

Lower middle-income countries have the second-lowest average income per person.

Upper middle-income countries have the second-highest average income per person.

High-income countries have the highest average income per person.

In 2019, what was the average income of people living in **low-income countries** and **lower middle-income countries**?

Please select the option you think is closest to the actual figures.

You will earn a **bonus of GBP 0.10** if your answer is correct.

- USD 1.00 per day for low-income country, USD 5.00 per day for lower middle-income country
- USD 2.00 per day for low-income country, USD 6.00 per day for lower middle-income country
- USD 3.00 per day for low-income country, USD 7.00 per day for lower middle-income country

In 2019, what was the share of the world's population living in **low-income countries** and **lower middle-income countries**?

Please select the option you think is closest to the actual figures.

You will earn a **bonus of GBP 0.10** if your answer is correct.

- The share of the world's population is the same in both low-income countries and lower-income countries
- The share of the world's population is higher in low-income countries than in lower middle-income countries
- The share of the world's population is higher in lower middle-income countries than in low-income countries

In 2019, what was the share of the world's extremely poor people living in **low-income countries** and **lower middle-income countries**?

Please select the option you think is closest to the actual figures.

You will earn a **bonus of GBP 0.10** if your answer is correct.

- The share of the world's extremely poor people is the same in both low-income countries and lower-income countries
- The share of the world's extremely poor people is higher in low-income countries than in lower middle-income countries
- The share of the world's extremely poor people is higher in lower middle-income countries than in low income countries

In 2017, what was the share of the world's vaccine-preventable deaths from infectious diseases (excluding COVID-19) in **low-income countries** and **lower middle-income countries**?

Please select the option you think is closest to the actual figures.

You will earn a **bonus of GBP 0.10** if your answer is correct.

- The share of the world's vaccine-preventable deaths is the same in both low-income countries and lower-income countries
- The share of the world's vaccine-preventable deaths is higher in low-income countries than in lower middle-income countries
- The share of the world's vaccine-preventable deaths is higher in lower middle-income countries than in low income countries

Gavi is an international organisation that aims to improve children's access to vaccines.

Are **low-income countries** eligible to receive Gavi support to access vaccines against infectious diseases (excluding COVID-19)?

Please select the option you think is closest to the actual eligibility status.

You will earn a **bonus of GBP 0.10** if your answer is correct.

- Yes
- No

Gavi is an international organisation that aims is to improve children's access to vaccines.

Are **lower middle-income countries** eligible to receive Gavi support to access vaccines against infectious diseases (excluding COVID-19)?

Please select the option you think is closest to the actual eligibility status.

You will earn a **bonus of GBP 0.10** if your answer is correct.

- Yes
- No

**End of Block: Pre-Survey Knowledge Quiz**

**Start of Block: Study Description and Quiz**

During a global pandemic, a key challenge is ensuring that a country has access to a vaccine. Vaccine access may partly depend on the price paid by a country to purchase vaccines from the producer.

The focus of this study is only on the price paid by a **country** to purchase the vaccines from the producer. After purchasing the vaccines, the government of a country may choose what price (if any) to charge its citizens.

Which of the following statements is correct?

You will earn a **bonus of GBP 0.10** if your answer is correct.

- This study only focuses on the price paid by a country to purchase the vaccines from the producer.
- This study only focuses on the price paid by the citizens within a country to purchase the vaccines.

Your answer is **INCORRECT**.

This study only focuses on the price paid by a **country** to purchase the vaccines from the producer.

Your answer is **CORRECT**. You have just earned an additional GBP 0.10.

This study only focuses on the price paid by a **country** to purchase the vaccines from the producer.

The total costs of vaccine production can be regarded as the **sum** of two types of costs.

- **Pre-production costs** are the costs of developing the vaccine and the production technology in the first place, **before any vaccines are produced for mass distribution.** Pre-production costs include the cost of research and development, conducting clinical trials, and constructing the manufacturing facility.
- **Production costs** are the costs of manufacturing the vaccine, **after a vaccine has been developed**. Production costs include the cost of operating the manufacturing facility and materials required to make the vaccines for mass distribution. Suppose a firm has developed a vaccine that has been approved by health authorities and developed the manufacturing capacity to supply the whole world, but **has not produced any vaccines yet**.

What type of costs has the firm incurred so far?

You will earn a **bonus of GBP 0.10** if your answer is correct.

- Production costs
- Pre-production costs

Your answer is **CORRECT**. You have just earned an additional GBP 0.10.

The firm has only incurred **pre-production costs** so far because it has not yet produced any vaccines for mass distribution.

Your answer is **INCORRECT**.

The firm has only incurred **pre-production costs** so far because it has not yet produced any vaccines for mass distribution.

Suppose a firm has developed a vaccine that has been approved by health authorities and developed the manufacturing capacity to supply the whole world.

The firm's expected **total cost**per dose is **USD 100**. The breakdown of the expected total cost per dose is as follows:

- Expected production cost per dose = **USD 10**
- Pre-production cost per dose = **USD 90**.

Suppose the firm can sell the vaccine at a price per dose of **USD 15**. Which of the following statements is correct?

You will earn a **bonus of GBP 0.10** if your answer is correct.

- The firm will be able to fully recover the expected production cost per dose of USD 10.
- The firm will not be able to fully recover the expected production cost per dose of USD 10.

Your answer is **CORRECT**. You have just earned an additional GBP 0.10.

The firm will be able to fully recover the expected production cost per dose of USD 10 because the firm can sell the vaccine at a price per dose of **USD 15**.

Your answer is **INCORRECT**.

The firm will be able to fully recover the expected production cost per dose of USD 10 because the firm can sell the vaccine at a price per dose of **USD 15**.

Suppose a firm has developed a vaccine that has been approved by health authorities and developed the manufacturing capacity to supply the whole world. 

The firm's expected **total cost**per dose is **USD 100**. The breakdown of the expected total cost per dose is as follows:

- Expected production cost per dose = **USD 10**
- Pre-production cost per dose = **USD 90**.

Suppose the firm sells the vaccines to a country for a price per dose of **USD 150**. Which of the following statements is correct?

You will earn a **bonus of GBP 0.10** if your answer is correct.

- The price per dose of USD 150 covers **none** of the expected total cost per dose (i.e. none of the production cost per dose and none of the pre-production cost per dose). The firm makes an overall loss per dose.
- The price per dose of USD 150 covers **some** of the expected production cost per dose, but **none** of the pre-production cost per dose. The firm makes an overall loss per dose.
- The price per dose of USD 150 covers **all** of the expected production cost per dose, but **none** of the pre-production cost per dose. The firm makes an overall loss per dose.
- The price per dose of USD 150 covers **all** of the expected production cost per dose, and **some** of the pre-production cost per dose. The firm makes an overall loss per dose.
- The price per dose of USD 150 covers **all** of the expected total cost per dose (i.e. all of the expected production cost per dose and all of the pre-production cost per dose). The firm breaks even and makes neither an overall profit nor overall loss per dose.
- The price per dose of USD 150 covers **more than** the expected total cost per dose. The firm makes some overall profit per dose.

Your answer is **CORRECT**. You have just earned an additional GBP 0.10.

The price per dose of USD 150 covers **more than** the expected total cost per dose (USD 100). The firm makes some overall profit per dose.

Your answer is **INCORRECT**.

The price per dose of USD 150 covers **more than** the expected total cost per dose (USD 100). The firm makes some overall profit per dose.

Suppose a firm has developed a vaccine that has been approved by health authorities and developed the manufacturing capacity to supply the whole world.

The firm's expected **total cost**per dose is **USD 100**. The breakdown of the expected total cost per dose is as follows:

- Expected production cost per dose = **USD 10**
- Pre-production cost per dose = **USD 90**.

Suppose the firm sells the vaccines to a country for a price per dose of **USD 100**. Which of the following statements is correct?

You will earn a **bonus of GBP 0.10** if your answer is correct.

- The price per dose of USD 100 covers **none** of the expected total cost per dose (i.e. none of the expected production cost per dose and none of the pre-production cost per dose). The firm makes an overall loss per dose.
- The price per dose of USD 100 covers **some** of the expected production cost per dose, but **none** of the pre-production cost per dose. The firm makes an overall loss per dose.
- The price per dose of USD 100 covers **all** of the expected production cost per dose, but **none** of the pre-production cost per dose. The firm makes an overall loss per dose.
- The price per dose of USD 100 covers **all** of the expected production cost per dose, and **some** of the pre-production cost per dose. The firm makes an overall loss per dose.
- The price per dose of USD 100 covers **all** of the expected total cost per dose (i.e. all of the production cost per dose and all of the pre-production cost per dose). The firm breaks even and makes neither an overall profit nor overall loss per dose.
- The price per dose of USD 100 covers **more than** the expected total cost per dose. The firm makes some overall profit per dose.

Your answer is **CORRECT**. You have just earned an additional GBP 0.10.

The price per dose of USD 100 covers **all** of the expected total cost per dose (USD 100) (i.e. all of the expected production cost per dose and all of the pre-production cost per dose). The firm breaks even and makes neither an overall profit nor overall loss per dose.

Your answer is **INCORRECT**.

The price per dose of USD 100 covers **all** of the expected total cost per dose (USD 100) (i.e. all of the expected production cost per dose and all of the pre-production cost per dose). The firm breaks even and makes neither an overall profit nor overall loss per dose.

Suppose a firm has developed a vaccine that has been approved by health authorities and developed the manufacturing capacity to supply the whole world.

The firm's expected **total cost**per dose is **USD 100**. The breakdown of the expected total cost per dose is as follows:

- Expected production cost per dose = **USD 10**
- Pre-production cost per dose = **USD 90**.

Suppose the firm sells the vaccines to a country for a price per dose of **USD 10**. Which of the following statements is correct?

You will earn a **bonus of GBP 0.10** if your answer is correct.

- The price per dose of USD 10 covers **none** of the expected total cost per dose (i.e. none of the expected production cost per dose and none of the pre-production cost per dose). The firm makes an overall loss per dose.
- The price per dose of USD 10 covers **some** of the expected production cost per dose, but **none** of the pre-production cost per dose. The firm makes an overall loss per dose.
- The price per dose of USD 10 covers **all** of the expected production cost per dose, but **none** of the pre-production cost per dose. The firm makes an overall loss per dose.
- The price per dose of USD 10 covers **all** of the expected production cost per dose, and **some** of the pre-production cost per dose. The firm makes an overall loss per dose.
- The price per dose of USD 10 covers **all** of the expected total cost per dose (i.e. all of the expected production cost per dose and all of the pre-production cost per dose). The firm makes neither an overall profit nor overall loss per dose.
- The price per dose of USD 10 covers **more than** the expected total cost per dose. The firm makes some overall profit per dose.

Your answer is **CORRECT**. You have just earned an additional GBP 0.10.

The price per dose of USD 10 covers **all** of the expected production cost per dose (USD 10), but **none** of the pre-production cost per dose (USD 90). The firm makes an overall loss per dose.

Your answer is **INCORRECT**.

The price per dose of USD 10 covers **all** of the expected production cost per dose (USD 10), but **none** of the pre-production cost per dose (USD 90). The firm makes an overall loss per dose.

Suppose a firm has developed a vaccine that has been approved by health authorities and developed the manufacturing capacity to supply the whole world.

The firm's expected **total cost**per dose is **USD 100**. The breakdown of the expected total cost per dose is as follows:

- Expected production cost per dose = **USD 10**
- Pre-production cost per dose = **USD 90**.

Suppose the firm sells the vaccines to a country for a price per dose of **USD 5**. Which of the following statements is correct?

You will earn a **bonus of GBP 0.10** if your answer is correct.

- The price per dose of USD 5 covers **none** of the expected total cost per dose (i.e. none of the expected production cost per dose and none of the pre-production cost per dose). The firm makes an overall loss per dose.
- The price per dose of USD 5 covers **some** of the expected production cost per dose, but **none** of the pre-production cost per dose. The firm makes an overall loss per dose.
- The price per dose of USD 5 covers **all** of the expected production cost per dose, but **none** of the pre-production cost per dose. The firm makes an overall loss per dose.
- The price per dose of USD 5 covers **all** of the expected production cost per dose, and **some** of the pre-production cost per dose. The firm makes an overall loss per dose.
- The price per dose of USD 5 covers **all** of the expected total cost per dose (i.e. all of the expected production cost per dose and all of the pre-production cost per dose). The firm breaks even and makes neither an overall profit nor overall loss per dose.
- The price per dose of USD 5 covers **more than** the expected total cost per dose. The firm makes some overall profit per dose.

Your answer is **CORRECT**. You have just earned an additional GBP 0.10.

The price per dose of USD 5 covers **some** of the expected production cost per dose (USD 10), but **none** of the pre-production cost per dose (USD 90). The firm makes an overall loss per dose.

Your answer is **INCORRECT**.

The price per dose of USD 5 covers **some** of the expected production cost per dose (USD 10), but **none** of the pre-production cost per dose (USD 90). The firm makes an overall loss per dose.

Suppose a firm has developed a vaccine that has been approved by health authorities and developed the manufacturing capacity to supply the whole world.

The firm's expected **total cost**per dose is **USD 100**. The breakdown of the expected total cost per dose is as follows:

- Expected production cost per dose = **USD 10**
- Pre-production cost per dose = **USD 90**.

Suppose the firm sells the vaccines to a country for a price per dose of **USD 50**. Which of the following statements is correct?

You will earn a **bonus of GBP 0.10** if your answer is correct.

- The price per dose of USD 50 covers **none** of the expected total cost per dose (i.e. none of the expected production cost per dose and none of the pre-production cost per dose). The firm makes an overall loss per dose.
- The price per dose of USD 50 covers **some** of the expected production cost per dose, but **none** of the pre-production cost per dose. The firm makes an overall loss per dose.
- The price per dose of USD 50 covers **all** of the expected production cost per dose, but **none** of the pre-production cost per dose. The firm makes an overall loss per dose.
- The price per dose of USD 50 covers **all** of the expected production cost per dose, and **some** of the pre-production cost per dose. The firm makes an overall loss per dose.
- The price per dose of USD 50 covers **all** of the expected total cost per dose (i.e. all of the expected production cost per dose and all of the pre-production cost per dose). The firm breaks even and makes neither an overall profit nor overall loss per dose.
- The price per dose of USD 50 covers **more than** the expected total cost per dose. The firm makes some overall profit per dose.

Your answer is **CORRECT**. You have just earned an additional GBP 0.10.

The price per dose of USD 50 covers **all** of the expected production cost per dose (USD 10), and **some** of the pre-production cost per dose (USD 90). The firm makes an overall loss per dose.

Your answer is **INCORRECT**.

The price per dose of USD 50 covers **all** of the expected production cost per dose (USD 10), and **some** of the pre-production cost per dose (USD 90). The firm makes an overall loss per dose.

A firm incurs both pre-production and production costs to produce a vaccine. To recover these costs and make a profit, the firm can charge the **same** price per dose to all countries. 

Alternatively, the firm can charge a **different price** per dose to different countries. For example, the firm can charge a higher price (above total cost) to country A, while charging a lower price (below total cost) to country B. This means the firm fully recovers costs and makes a **profit from country A**, but does not fully recover total costs and makes a **loss from country B**. The firm's total profits equal to the **sum of the profits** from country A and country B.

True or False: A firm that charges a **different price** per dose to different countries makes a **different profit/loss** per dose from different countries.

You will earn a **bonus of GBP 0.10** if your answer is correct.

- True
- False

Your answer is **CORRECT**. You have just earned an additional GBP 0.10.

The correct answer is **TRUE**. A firm that charges a **different price per dose**to different countries makes a **different profit/loss per dose** from different countries.

Your answer is **INCORRECT**.

The correct answer is **TRUE**. A firm that charges a **different price per dose**to different countries makes a **different profit/loss per dose** from different countries.

To check your understanding, we will now ask you some questions relating to a **different**scenario.

Suppose a firm has developed a vaccine that has been approved by health authorities and developed the manufacturing capacity to supply the whole world.

The firm's expected **total cost** per dose is **USD 200**. The breakdown of the expected total cost per dose is as follows:

- Expected production cost per dose = **USD 20**
- Pre-production cost per dose = **USD 180**.

Suppose the firm sells the vaccine to a country for a price per dose of **USD 20**. Which of the following statements is correct?

You will earn a **bonus of GBP 0.10** if your answer is correct.

- The price per dose of USD 20 covers **none** of the expected total cost per dose (i.e. none of the expected production cost per dose and none of the pre-production cost per dose). The firm makes an overall loss per dose.
- The price per dose of USD 20 covers **some** of the expected production cost per dose, but **none** of the pre-production cost per dose. The firm makes an overall loss per dose.
- The price per dose of USD 20 covers **all** of the expected production cost per dose, but **none** of the pre-production cost per dose. The firm makes an overall loss per dose.
- The price per dose of USD 20 covers **all** of the expected production cost per dose, and **some** of the pre-production cost per dose. The firm makes an overall loss per dose.
- The price per dose of USD 20 covers **all** of the expected total cost per dose (i.e. all of the expected production cost per dose and all of the pre-production cost per dose. The firm makes neither an overall profit nor overall loss per dose.
- The price per dose of USD 20 covers **more than**the expected total cost per dose. The firm makes some overall profit per dose.

Your answer is **CORRECT**. You have just earned an additional GBP 0.10.

The price per dose of USD 20 covers **all** of the expected production cost per dose (USD 20), but **none** of the pre-production cost per dose (USD 180). The firm makes an overall loss per dose.

Your answer is **INCORRECT**.

The price per dose of USD 20 covers **all** of the expected production cost per dose (USD 20), but **none** of the pre-production cost per dose (USD 180). The firm makes an overall loss per dose.

Suppose a firm has developed a vaccine that has been approved by health authorities and developed the manufacturing capacity to supply the whole world.

The firm's expected **total cost** per dose is **USD 200**. The breakdown of the expected total cost per dose is as follows:

- Expected production cost per dose = **USD 20**
- Pre-production cost per dose = **USD 180**.

Suppose the firm sells the vaccine to a country for a price per dose of **USD 10**. Which of the following statements is correct?

You will earn a **bonus of GBP 0.10** if your answer is correct.

- The price per dose of USD 10 covers **none** of the expected total cost per dose (i.e. none of the expected production cost per dose and none of the pre-production cost per dose). The firm makes an overall loss per dose.
- The price per dose of USD 10 covers **some** of the expected production cost per dose, but **none** of the pre-production cost per dose. The firm makes an overall loss per dose.
- The price per dose of USD 10 covers **all** of the expected production cost per dose, but **none** of the pre-production cost per dose. The firm makes an overall loss per dose.
- The price per dose of USD 10 covers **all** of the expected production cost per dose, and **some** of the pre-production cost per dose. The firm makes an overall loss per dose.
- The price per dose of USD 10 covers **all** of the expected total cost per dose (i.e. all of the expected production cost per dose and all of the pre-production cost per dose. The firm makes neither an overall profit nor overall loss per dose.
- The price per dose of USD 10 covers **more than**the expected total cost per dose. The firm makes some overall profit per dose.

Your answer is **CORRECT**. You have just earned an additional GBP 0.10.

The price per dose of USD 10 covers **some** of the expected production cost per dose (USD 20), but **none** of the pre-production cost per dose (USD 180). The firm makes an overall loss per dose.

Your answer is **INCORRECT**.

The price per dose of USD 10 covers **some** of the expected production cost per dose (USD 20), but **none** of the pre-production cost per dose (USD 180). The firm makes an overall loss per dose.

Suppose a firm has developed a vaccine that has been approved by health authorities and developed the manufacturing capacity to supply the whole world.

The firm's expected **total cost** per dose is **USD 200**. The breakdown of the expected total cost per dose is as follows:

- Expected production cost per dose = **USD 20**
- Pre-production cost per dose = **USD 180**.

Suppose the firm sells the vaccine to a country for a price per dose of **USD 25**. Which of the following statements is correct?

You will earn a **bonus of GBP 0.10** if your answer is correct.

- The price per dose of USD 25 covers **none** of the expected total cost per dose (i.e. none of the expected production cost per dose and none of the pre-production cost per dose). The firm makes an overall loss per dose.
- The price per dose of USD 25 covers **some** of the expected production cost per dose, but **none** of the pre-production cost per dose. The firm makes an overall loss per dose.
- The price per dose of USD 25 covers **all** of the expected production cost per dose, but **none** of the pre-production cost per dose. The firm makes an overall loss per dose.
- The price per dose of USD 25 covers **all** of the expected production cost per dose, and **some** of the pre-production cost per dose. The firm makes an overall loss per dose.
- The price per dose of USD 25 covers **all** of the expected total cost per dose (i.e. all of the expected production cost per dose and all of the pre-production cost per dose. The firm makes neither an overall profit nor overall loss per dose.
- The price per dose of USD 25 covers **more than**the expected total cost per dose. The firm makes some overall profit per dose.

Your answer is **CORRECT**. You have just earned an additional GBP 0.10.

The price per dose of USD 25 covers **all** of the expected production cost per dose (USD 20), and **some** of the pre-production cost per dose (USD 180). The firm makes an overall loss per dose.

Your answer is **INCORRECT**.

The price per dose of USD 25 covers **all** of the expected production cost per dose (USD 20), and **some** of the pre-production cost per dose (USD 180). The firm makes an overall loss per dose.

**End of Block: Study Description and Quiz**

**Start of Block: Information Treatment 1 Preamble**

We will now present some information on low, lower-middle, upper-middle and high income countries that may help you answer the survey questions.

**End of Block: Information Treatment 1 Preamble**

**Start of Block: Information Treatment 1 (facts)**

According to the World Bank, the average income of people across all countries around the world is as follows:


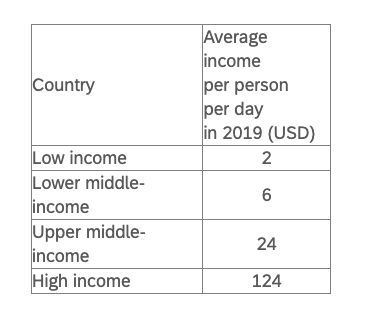


Based on the above description, which of the following statements is correct?

- Low-income countries have an average income of USD 2 per person per day.
- Lower middle-income countries have an average income of USD 2 per person per day.
- Upper middle-income countries have an average income of USD 2 per person per day.
- High-income countries have an average income of USD 2 per person per day.

According to the World Bank and Institute for Health Metrics and Evaluation, the distribution of the world's population, extremely poor people, and vaccine-preventable deaths is given by the table below.


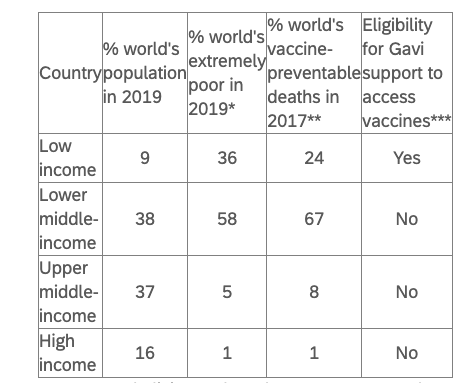


*Note: people living on less than USD 1.90 per day

**Note: several infectious diseases excluding COVID-19

***Note: support includes vaccines in-kind and financial support

Gavi is an international organisation that aims to improve access to vaccines for children living in the world's poorest countries. Currently, only low-income countries are eligible for Gavi support to access vaccines, although they are still required to contribute to the cost of vaccines.

Based on the above description, which of the following statements is correct?

- Low-income countries have the majority of the world's extremely poor people and vaccine-preventable deaths, and are not eligible for Gavi support to access vaccines.
- Lower middle-income countries have the majority of the world's extremely poor people and vaccine-preventable deaths, and are not eligible for Gavi support to access vaccines.
- Upper middle-income countries have the majority of the world's extremely poor people and vaccine-preventable deaths, and are not eligible for Gavi support to access vaccines.
- High-income countries have the majority of the world's extremely poor people and vaccine-preventable deaths, and are not eligible for Gavi support to access vaccines.

According to estimates from the World Health Organization, researchers and industry, some characteristics of the development and global market value of selected pharmaceutical products are as follows:


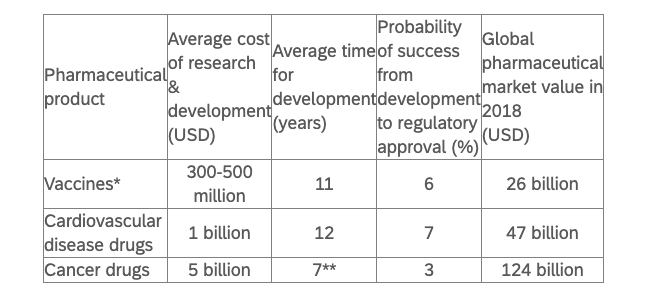


*Note: several infectious diseases excluding COVID-19

**Note: median

Based on the above description, which of the following statements is correct?

- Cardiovascular disease drugs had the lowest global pharmaceutical market value in 2018.
- Cancer drugs had the lowest global pharmaceutical market value in 2018.
- Vaccines had the lowest global pharmaceutical market value in 2018.

**End of Block: Information Treatment 1 (facts)**

**Start of Block: Information Treatment 1 (feedback)**

Your answer is **CORRECT**.

Low-income countries have an average income of USD 2 per person per day.

Lower middle-income countries have an average income of USD 6 per person per day.

Upper middle-income countries have an average income of USD 24 per person per day.

High-income countries have an average income of USD 124 per person per day.

Your answer is **INCORRECT**.

Low-income countries have an average income of USD 2 per person per day.

Lower middle-income countries have an average income of USD 6 per person per day.

Upper middle-income countries have an average income of USD 24 per person per day.

High-income countries have an average income of USD 124 per person per day.

Your answer is **CORRECT**.

Lower middle-income countries have the majority of the world's extremely poor people and vaccine-preventable deaths, and are not eligible for Gavi support to access vaccines.

Your answer is **INCORRECT**.

Lower middle-income countries have the majority of the world's extremely poor people and vaccine-preventable deaths, and are not eligible for Gavi support to access vaccines.

Your answer is **CORRECT**.

Vaccines had the lowest global pharmaceutical market value in 2018.

Your answer is **INCORRECT**.

Vaccines had the lowest global pharmaceutical market value in 2018.

**End of Block: Information Treatment 1 (feedback)**

**Start of Block: Information Treatment 2 Preamble**

We will now present some factors that have been considered in the pricing of vaccines across different countries.

These factors may help you think about the global pricing of vaccines and answer some questions that we will ask you.

**End of Block: Information Treatment 2 Preamble**

**Start of Block: Information Treatment 2 (factors without fact refs)**

**Factor:** The overall pricing strategy of vaccines across different countries should aim to make the vaccine affordable for all countries and be based on a country's **ability to pay**.

It has been argued that vaccine pricing should ensure that low-income countries pay the lowest price.

How strongly do you agree or disagree with the following statement?

"High-income countries should pay the most for vaccines."

- Strongly disagree
- Disagree
- Neither agree nor disagree
- Agree
- Strongly agree

**Factor**: The overall pricing strategy of vaccines across different countries should aim to make vaccines available to countries that **need** them the most as quickly as possible.

It has been argued that vaccine pricing should ensure that lower middle-income countries have adequate access to vaccines.

How strongly do you agree or disagree with the following statement?

"Lower middle-income countries should receive adequate support to access vaccines, and pay a price for vaccines that allows them to prevent deaths and reduce the burden of extreme poverty."

- Strongly disagree
- Disagree
- Neither agree nor disagree
- Agree
- Strongly agree

**Factor**: Firms should have sufficient **incentive to continue to research, develop, innovate and supply** vaccines.

It has been argued that the combination of government support and vaccine pricing should ensure that firms are appropriately encouraged and rewarded for investment in vaccine research and development.

How strongly do you agree or disagree with the following statement?

"The combination of government support and vaccine pricing should give firms sufficient incentive to continue to research, develop, innovate and supply vaccines to meet global health needs."

- Strongly disagree
- Disagree
- Neither agree nor disagree
- Agree
- Strongly agree

**End of Block: Information Treatment 2 (factors without fact refs)**

**Start of Block: Information Treatment 3 Preamble**

We will now present some factors that have been considered in the pricing of vaccines across different countries.

These factors may help you think about the global pricing of vaccines and answer some questions that we will ask you.

**End of Block: Information Treatment 3 Preamble**

**Start of Block: Information Treatment 3 (factors with fact refs)**

**Factor:** The overall pricing strategy of vaccines across different countries should aim to make the vaccine affordable for all countries and be based on a country's **ability to pay**.

It has been argued that vaccine pricing should ensure that low-income countries pay the lowest price because they have the lowest average income.

How strongly do you agree or disagree with the following statement?

"High-income countries should pay the most for vaccines."

- Strongly disagree
- Disagree
- Neither agree nor disagree
- Agree
- Strongly agree

**Factor**: The overall pricing strategy of vaccines across different countries should aim to make vaccines available to countries that **need** them the most as quickly as possible.

It has been argued that vaccine pricing should ensure that lower middle-income countries have adequate access to vaccines because they are not eligible for Gavi support to access

vaccines, despite having the majority of the world's extremely poor people and vaccine-preventable deaths.

How strongly do you agree or disagree with the following statement?

"Lower middle-income countries should receive adequate support to access vaccines, and pay a price for vaccines that allows them to prevent deaths and reduce the burden of extreme poverty."

- Strongly disagree
- Disagree
- Neither agree nor disagree
- Agree
- Strongly agree

**Factor**: Firms should have sufficient **incentive to continue to research, develop, innovate and supply** vaccines.

It has been argued that the combination of government support and vaccine pricing should ensure that firms are appropriately encouraged and rewarded for costly and risky investment

in vaccine research and development, because it usually takes many years and has a high failure rate, but has low global market value.

How strongly do you agree or disagree with the following statement?

"The combination of government support and vaccine pricing should give firms sufficient incentive to continue to research, develop, innovate and supply vaccines to meet global health needs."

- Strongly disagree
- Disagree
- Neither agree nor disagree
- Agree
- Strongly agree

**End of Block: Information Treatment 3 (factors with fact refs)**

**Start of Block: Personal Choice Questions Preamble**

This is the first part of the main survey.

You will be asked some questions related to the pricing of vaccines across different countries.

**End of Block: Personal Choice Questions Preamble**

**Start of Block: Personal Choice Questions (Low TC, Low MC)**

Suppose that during a global pandemic, a firm has developed a safe and effective vaccine that has been approved by health authorities and developed the manufacturing capacity to supply the whole world.

The firm can charge either the same or different price per dose across different countries, and therefore make either the same or different profit/loss per dose across different countries.

The expected **total cost** per dose is **USD 10.** The breakdown of the expected total cost per dose is as follows:

- Expected production cost per dose = **USD 3**
- Pre-production cost per dose = **USD 7**

In your view, what price per dose should the firm charge a **low-income country**?

Please select one of the following options.

- **Less than USD 3**. The firm makes an overall loss per dose.
- **Equal to USD 3**. The firm makes an overall loss per dose.
- **More than USD 3 but less than USD 6.50**. The firm makes an overall loss per dose.
- **More than USD 6.50 but less than USD 10**. The firm makes an overall loss per dose.
- **Equal to USD 10**. The firm breaks even and makes neither an overall profit nor overall loss per dose.
- **More than USD 10**. The firm makes some overall profit per dose.

The expected **total cost** per dose is **USD 10.** The breakdown of the expected total cost per dose is as follows:

- Expected production cost per dose = **USD 3**
- Pre-production cost per dose = **USD 7**

In your view, what price per dose should the firm charge a **lower middle-income country**?

Please select one of the following options.

- **Less than USD 3**. The firm makes an overall loss per dose.
- **Equal to USD 3.** The firm makes an overall loss per dose.
- **More than USD 3 but less than USD 6.50**. The firm makes an overall loss per dose.
- **More than USD 6.50 but less than USD 10**. The firm makes an overall loss per dose.
- **Equal to USD 10**. The firm breaks even and makes neither an overall profit nor overall loss per dose.
- **More than USD 10**. The firm makes some overall profit per dose.

The expected **total cost** per dose is **USD 10.** The breakdown of the expected total cost per dose is as follows:

- Expected production cost per dose = **USD 3**
- Pre-production cost per dose = **USD 7**

In your view, what price per dose should the firm charge an **upper middle-income country**?

Please select one of the following options.

- **Less than USD 3**. The firm makes an overall loss per dose.
- **Equal to USD 3**. The firm makes an overall loss per dose.
- **More than USD 3 but less than USD 6.50**. The firm makes an overall loss per dose.
- **More than USD 6.50 but less than USD 10**. The firm makes an overall loss per dose.
- **Equal to USD 10**. The firm breaks even and makes neither an overall profit nor overall loss per dose.
- **More than USD 10**. The firm makes some overall profit per dose.

The expected **total cost** per dose is **USD 10.** The breakdown of the expected total cost per dose is as follows:

- Expected production cost per dose = **USD 3**
- Pre-production cost per dose = **USD 7**

In your view, what price per dose should the firm charge a **high-income country**?

Please select one of the following options.

- **Less than USD 3**. The firm makes an overall loss per dose.
- **Equal to USD 3**. The firm makes an overall loss per dose.
- **More than USD 3 but less than USD 6.50**. The firm makes an overall loss per dose.
- **More than USD 6.50 but less than USD 10**. The firm makes an overall loss per dose.
- **Equal to USD 10**. The firm breaks even and makes neither an overall profit nor overall loss per dose.
- **More than USD 10**. The firm makes some overall profit per dose.

In this scenario, the expected **total cost**per dose is **USD 10**. The breakdown of the expected total cost per dose is as follows:

- Expected production cost per dose = **USD 3**
- Pre-production cost per dose = **USD 7**

In your view, should the firm make some overall profit from its pricing across different countries?

Please select one of the following options.

- Yes, the firm should make at least some overall profit
- Unsure
- No, the firm should make no overall profit

**End of Block: Personal Choice Questions (Low TC, Low MC)**

**Start of Block: Co-ordination Questions Preamble and Quiz**

This is the second part of the main survey.

You will now be asked the **exact same questions that you just completed**.

Again, you will have to choose:

- the price per dose for a type of country
- whether the firm should make some overall profit or not.

But there is one important difference.

You will earn a **bonus of GBP 0.10** for each question if the option you select is the option **chosen most frequently among all of the survey participants** that represent the US population in terms of age, sex, race and education.

Therefore, when making your choices in each scenario, you should ask yourself:

**What is the price per dose for this type of country that is most likely to be chosen by the other participants?**

**Would the other participants most likely choose that the firm should make some overall profit or not?**

Before proceeding, please answer the following question.

Which of the following **TWO** statements are correct?

- I will earn a bonus of GBP 0.10 regardless of my choice in each scenario.
- I will not earn anything regardless of my choice in each scenario.
- I will earn a bonus of GBP 0.10 if my choice regarding the price per dose for a type of country is also the choice that is selected most frequently among all of the survey participants in each scenario.
- I will earn a bonus of GBP 0.10 if my choice regarding whether or not the firm should make some overall profit is also the choice that is selected most frequently among all of the survey participants in each scenario.

Your answer is **CORRECT**.

You will earn a bonus of GBP 0.10 if your choice regarding the price per dose for a type of country is also the choice that is selected most frequently among all of the survey participants in each scenario.

You will earn a bonus of GBP 0.10 if your choice regarding whether or not the firm should make some overall profit is also the choice that is selected most frequently among all of the survey participants in each scenario.

Your answer is **INCORRECT**.

You will earn a bonus of GBP 0.10 if your choice regarding the price per dose for a type of country is also the choice that is selected most frequently among all of the survey participants in each scenario.

You will earn a bonus of GBP 0.10 if your choice regarding whether or not the firm should make some overall profit is also the choice that is selected most frequently among all of the survey participants in each scenario.

**End of Block: Co-ordination Questions Preamble and Quiz**

**Start of Block: Co-ordination Questions (Low TC, Low MC)**

Suppose that during a global pandemic, a firm has developed a safe and effective vaccine that has been approved by health authorities and developed the manufacturing capacity to supply the whole world.

The firm can charge either the same or different price per dose across different countries, and therefore make either the same or different profit/loss per dose across different countries.

The expected **total cost** per dose is **USD 10.** The breakdown of the expected total cost per dose is as follows:

- Expected production cost per dose = **USD 3**
- Pre-production cost per dose = **USD 7**

What price per dose should the firm charge a **low-income country**?

Please select one of the following options.

You will earn a **bonus of GBP 0.10** if the option you select is the option **chosen most frequently among all of the survey participants** that represent the US population in terms of age, sex, race and education.

- **Less than USD 3**. The firm makes an overall loss per dose.
- **Equal to USD 3**. The firm makes an overall loss per dose.
- **More than USD 3 but less than USD 6.50**. The firm makes an overall loss per dose.
- **More than USD 6.50 but less than USD 10**. The firm makes an overall loss per dose.
- **Equal to USD 10**. The firm breaks even and makes neither an overall profit nor overall loss per dose.
- **More than USD 10**. The firm makes some overall profit per dose.

The expected **total cost** per dose is **USD 10.** The breakdown of the expected total cost per dose is as follows:

- Expected production cost per dose = **USD 3**
- Pre-production cost per dose = **USD 7**

What price per dose should the firm charge a **lower middle-income country**?

Please select one of the following options.

You will earn a **bonus of GBP 0.10** if the option you select is the option **chosen most frequently among all of the survey participants** that represent the US population in terms of age, sex, race and education.

- **Less than USD 3**. The firm makes an overall loss per dose.
- **Equal to USD 3**. The firm makes an overall loss per dose.
- **More than USD 3 but less than USD 6.50**. The firm makes an overall loss per dose.
- **More than USD 6.50 but less than USD 10**. The firm makes an overall loss per dose.
- **Equal to USD 10**. The firm breaks even and makes neither an overall profit nor overall loss per dose.
- **More than USD 10**. The firm makes some overall profit per dose.

The expected **total cost** per dose is **USD 10.** The breakdown of the expected total cost per dose is as follows:

- Expected production cost per dose = **USD 3**
- Pre-production cost per dose = **USD 7**

What price per dose should the firm charge an **upper middle-income country**?

Please select one of the following options.

You will earn a **bonus of GBP 0.10** if the option you select is the option **chosen most frequently among all of the survey participants** that represent the US population in terms of age, sex, race and education.

- **Less than USD 3**. The firm makes an overall loss per dose.
- **Equal to USD 3**. The firm makes an overall loss per dose.
- **More than USD 3 but less than USD 6.50**. The firm makes an overall loss per dose.
- **More than USD 6.50 but less than USD 10**. The firm makes an overall loss per dose.
- **Equal to USD 10**. The firm breaks even and makes neither an overall profit nor overall loss per dose.
- **More than USD 10**. The firm makes some overall profit per dose.

The expected **total cost** per dose is **USD 10.** The breakdown of the expected total cost per dose is as follows:

- Expected production cost per dose = **USD 3**
- Pre-production cost per dose = **USD 7**

What price per dose should the firm charge a **high-income country**?

Please select one of the following options.

You will earn a **bonus of GBP 0.10** if the option you select is the option **chosen most frequently among all of the survey participants** that represent the US population in terms of age, sex, race and education.

- **Less than USD 3**. The firm makes an overall loss per dose.
- **Equal to USD 3**. The firm makes an overall loss per dose.
- **More than USD 3 but less than USD 6.50**. The firm makes an overall loss per dose.
- **More than USD 6.50 but less than USD 10**. The firm makes an overall loss per dose.
- **Equal to USD 10**. The firm breaks even and makes neither an overall profit nor overall loss per dose.
- **More than USD 10**. The firm makes some overall profit per dose.

In this scenario, the expected **total cost**per dose is **USD 10**. The breakdown of the expected total cost per dose is as follows:

- Expected production cost per dose = **USD 3**
- Pre-production cost per dose = **USD 7**

Should the firm make some overall profit from its pricing across different countries?

Please select one of the following options.

You will earn a **bonus of GBP 0.10** if the option you select is the option **chosen most frequently among all of the survey participants** that represent the US population in terms of age, sex, race and education.

- Yes, the firm should make at least some overall profit
- Unsure
- No, the firm should make no overall profit

**End of Block: Co-ordination Questions (Low TC, Low MC)**

**Start of Block: Personal Choice Questions (High TC, Low MC)**

Suppose that during a global pandemic, a firm has developed a safe and effective vaccine that has been approved by health authorities and developed the manufacturing capacity to supply the whole world.

The firm can charge either the same or different price per dose across different countries, and therefore make either the same or different profit/loss per dose across different countries.

The expected **total cost** per dose is **USD 50.** The breakdown of the expected total cost per dose is as follows:

- Expected production cost per dose = **USD 15**
- Pre-production cost per dose = **USD 35**

In your view, what price per dose should the firm charge a **low-income country**?

Please select one of the following options.

- **Less than USD 15**. The firm makes an overall loss per dose.
- **Equal to USD 15**. The firm makes an overall loss per dose.
- **More than USD 15 but less than USD 32.50**. The firm makes an overall loss per dose.
- **More than USD 32.50 but less than USD 50**. The firm makes an overall loss per dose.
- **Equal to USD 50.** The firm breaks even and makes neither an overall profit nor overall loss per dose.
- **More than USD 50**. The firm makes some overall profit per dose.

The expected **total cost** per dose is **USD 50.** The breakdown of the expected total cost per dose is as follows:

- Expected production cost per dose = **USD 15**
- Pre-production cost per dose = **USD 35**

In your view, what price per dose should the firm charge a **lower middle-income country**?

Please select one of the following options.

- **Less than USD 15.** The firm makes an overall loss per dose.
- **Equal to USD 15**. The firm makes an overall loss per dose.
- **More than USD 15 but less than USD 32.50**. The firm makes an overall loss per dose.
- **More than USD 32.50 but less than USD 50**. The firm makes an overall loss per dose.
- **Equal to USD 50**. The firm breaks even and makes neither an overall profit nor overall loss per dose.
- **More than USD 50**. The firm makes some overall profit per dose.

The expected **total cost** per dose is **USD 50.** The breakdown of the expected total cost per dose is as follows:

- Expected production cost per dose = **USD 15**
- Pre-production cost per dose = **USD 35**

In your view, what price per dose should the firm charge an **upper middle-income country**?

Please select one of the following options.

- **Less than USD 15.** The firm makes an overall loss per dose.
- **Equal to USD 15**. The firm makes an overall loss per dose.
- **More than USD 15 but less than USD 32.50**. The firm makes an overall loss per dose.
- **More than USD 32.50 but less than USD 50**. The firm makes an overall loss per dose.
- **Equal to USD 50**. The firm breaks even and makes neither an overall profit nor overall loss per dose.
- **More than USD 50**. The firm makes some overall profit per dose.

The expected **total cost** per dose is **USD 50.** The breakdown of the expected total cost per dose is as follows:

- Expected production cost per dose = **USD 15**
- Pre-production cost per dose = **USD 35**

In your view, what price per dose should the firm charge a **high-income country**?

Please select one of the following options.

- **Less than USD 15**. The firm makes an overall loss per dose.
- **Equal to USD 15**. The firm makes an overall loss per dose.
- **More than USD 15 but less than USD 32.50**. The firm makes an overall loss per dose.
- **More than USD 32.50 but less than USD 50**. The firm makes an overall loss per dose.
- **Equal to USD 50**. The firm breaks even and makes neither an overall profit nor overall loss per dose.
- **More than USD 50**. The firm makes some overall profit per dose.

In this scenario, the expected **total cost**per dose is **USD 50**. The breakdown of the expected total cost per dose is as follows:

- Expected production cost per dose = **USD 15**
- Pre-production cost per dose = **USD 35**

In your view, should the firm make some overall profit from its pricing across different countries?

Please select one of the following options.

- Yes, the firm should make at least some overall profit
- Unsure
- No, the firm should make no overall profit

**End of Block: Personal Choice Questions (High TC, Low MC)**

**Start of Block: Co-ordination Questions (High TC, Low MC)**

Suppose that during a global pandemic, a firm has developed a safe and effective vaccine that has been approved by health authorities and developed the manufacturing capacity to supply the whole world.

The firm can charge either the same or different price per dose across different countries, and therefore make either the same or different profit/loss per dose across different countries.

The expected **total cost** per dose is **USD 50.** The breakdown of the expected total cost per dose is as follows:

- Expected production cost per dose = **USD 15**
- Pre-production cost per dose = **USD 35**

What price per dose should the firm charge a **low-income country**?

Please select one of the following options.

You will earn a **bonus of GBP 0.10** if the option you select is the option **chosen most frequently among all of the survey participants** that represent the US population in terms of age, sex, race and education.

- **Less than USD 15**. The firm makes an overall loss per dose.
- **Equal to USD 15**. The firm makes an overall loss per dose.
- **More than USD 15 but less than USD 32.50**. The firm makes an overall loss per dose.
- **More than USD 32.50 but less than USD 50**. The firm makes an overall loss per dose.
- **Equal to USD 50**. The firm breaks even and makes neither an overall profit nor overall loss per dose.
- **More than USD 50**. The firm makes some overall profit per dose.

The expected **total cost** per dose is **USD 50.** The breakdown of the expected total cost per dose is as follows:

- Expected production cost per dose = **USD 15**
- Pre-production cost per dose = **USD 35**

What price per dose should the firm charge a **lower middle- income country**?

Please select one of the following options.

You will earn a **bonus of GBP 0.10** if the option you select is the option **chosen most frequently among all of the survey participants** that represent the US population in terms of age, sex, race and education.

- **Less than USD 15**. The firm makes an overall loss per dose.
- **Equal to USD 15**. The firm makes an overall loss per dose.
- **More than USD 15 but less than USD 32.50**. The firm makes an overall loss per dose.
- **More than USD 32.50 but less than USD 50**. The firm makes an overall loss per dose.
- **Equal to USD 50**. The firm breaks even and makes neither an overall profit nor overall loss per dose.
- **More than USD 50**. The firm makes some overall profit per dose.

The expected **total cost** per dose is **USD 50.** The breakdown of the expected total cost per dose is as follows:

- Expected production cost per dose = **USD 15**
- Pre-production cost per dose = **USD 35**

What price per dose should the firm charge an **upper middle-income country**?

Please select one of the following options.

You will earn a **bonus of GBP 0.10** if the option you select is the option **chosen most frequently among all of the survey participants** that represent the US population in terms of age, sex, race and education.

- **Less than USD 15**. The firm makes an overall loss per dose.
- **Equal to USD 15**. The firm makes an overall loss per dose.
- **More than USD 15 but less than USD 32.50**. The firm makes an overall loss per dose.
- **More than USD 32.50 but less than USD 50**. The firm makes an overall loss per dose.
- **Equal to USD 50**. The firm breaks even and makes neither an overall profit nor overall loss per dose.
- **More than USD 50**. The firm makes some overall profit per dose.

The expected **total cost** per dose is **USD 50.** The breakdown of the expected total cost per dose is as follows:

- Expected production cost per dose = **USD 15**
- Pre-production cost per dose = **USD 35**

What price per dose should the firm charge a **high-income country**?

Please select one of the following options.

You will earn a **bonus of GBP 0.10** if the option you select is the option **chosen most frequently among all of the survey participants** that represent the US population in terms of age, sex, race and education.

- **Less than USD 15**. The firm makes an overall loss per dose.
- **Equal to USD 15**. The firm makes an overall loss per dose.
- **More than USD 15 but less than USD 32.50**. The firm makes an overall loss per dose.
- **More than USD 32.50 but less than USD 50**. The firm makes an overall loss per dose.
- **Equal to USD 50**. The firm breaks even and makes neither an overall profit nor overall loss per dose.
- **More than USD 50**. The firm makes some overall profit per dose.

In this scenario, the expected **total cost**per dose is **USD 50**. The breakdown of the expected total cost per dose is as follows:

- Expected production cost per dose = **USD 15**
- Pre-production cost per dose = **USD 35**

Should the firm make some overall profit from its pricing across different countries?

Please select one of the following options.

You will earn a **bonus of GBP 0.10** if the option you select is the option **chosen most frequently among all of the survey participants** that represent the US population in terms of age, sex, race and education.

- Yes, the firm should make at least some overall profit
- Unsure
- No, the firm should make no overall profit

**End of Block: Co-ordination Questions (High TC, Low MC)**

**Start of Block: Demographic Questions (US)**

The main part of the survey is now over. We will now ask you some general questions about yourself.

How would you rate the difficulty of completing this survey?

- Very difficult
- Difficult
- Fair
- Easy
- Very easy

What is your age in years?

________________________________________________________________

What is your sex?

- Male
- Female

Which state do you currently live in?

________________________________________________________________

Which city or town do you currently live in?

________________________________________________________________

Are you of Hispanic, Latino, or Spanish origin?

- No, not of Hispanic, Latino, or Spanish origin
- Yes, Mexican, Mexican American, Chicano
- Yes, Puerto Rican
- Yes, Cuban
- Yes, another Hispanic, Latino or Spanish origin. Please specify:

________________________________________________

What is your race?

- White (including Hispanic, Latino or Spanish origin)
- Black or African American
- Native American Indian or Alaska Native
- Asian or Asian American
- Native Hawaiian or Other Pacific Islander
- Other, please specify:

________________________________________________

What religious denomination do you belong to or identify most closely with?

- Protestant (Baptist, Methodist, Non-denominational, Lutheran, Presbyterian, Pentecostal, Episcopalian, Reformed, Church of Christ etc.)
- Roman Catholic (Catholic)
- Mormon (Church of Jesus Christ of Latter-day Saints/LDS)
- Orthodox (Greek, Russian, or some other orthodox church)
- Jewish
- Muslim
- Other religion, please specify: ________________________________________________
- Atheist
- Agnostic
- No religion

How important is religion in your life?

- Not important at all
- Somewhat important
- Very important
- Extremely important

What is the highest level of education you have completed?

- Did not finish high school (no high school diploma)
- Finished high school (high school diploma or the equivalent, e.g. GED)
- Some years of college or university (no degree)
- Completed Associate's or Bachelor's degree
- More than Bachelor's degree (e.g. Master's, Doctorate, Professional degree)

In current politics, which category do you consider yourself more of?

- Democrat
- Republican
- Independent, leaning towards Democrat
- Independent, leaning towards Republican
- Independent/Other, please specify:

________________________________________________

**End of Block: Demographic Questions (US)**

**Start of Block: CRT Questions**

Before we finish, we will ask you some questions that are not related to the main survey.

You will earn a **bonus of GBP 0.10** if your answer is correct.

Jerry received both the 15th highest and the 15th lowest mark in the class. How many students are in the class?

You will earn a **bonus of GBP 0.10** if your answer is correct.

- 28
- 29
- 30
- 31
- 32

A man buys a pig for $60, sells it for $70, buys it back for $80, and sells it finally for $90. How much has he made?

You will earn a **bonus of GBP 0.10** if your answer is correct.

- $0
- $10
- $20
- $30
- $40

If you are running a race and you pass the person in second place, what place are you in?

You will earn a **bonus of GBP 0.10** if your answer is correct.

- first
- second
- third
- fourth
- last

If John can drink one barrel of water in 6 days, and Mary can drink one barrel of water in 12 days, how many days would it take them to drink one barrel of water together?

You will earn a **bonus of GBP 0.10** if your answer is correct.

- 2
- 4
- 6
- 9
- 12

A bat and a ball cost a total of USD 220. The bat costs USD 200 more than the ball. What is the cost of the ball?

You will earn a **bonus of GBP 0.10** if your answer is correct.

- USD 5
- USD 10
- USD 20
- USD 200
- USD 210

Thank you for participating in this study.

Please click NEXT to finish.

**End of Block: CRT Questions**

**References**

1. Slunge D. The Willingness to Pay for Vaccination against Tick-Borne Encephalitis and Implications for Public Health Policy: Evidence from Sweden*. PLoS One* 2015;**10**(2): e0143975. doi: 10.1371/journal.pone.0143875

2. Costa-Font J, Ruisill C, Harrison S, *et al*. The Social Value of a SARS-CoV-2 Vaccine: Willingness to Pay Estimates from Four Western Countries. IZA Discussion Paper No. 14475. June 2021. Available: http://ftp.iza.org/dp14475.pdf [Accessed 26 Jul 2021]

3. Cerda AA, Garcia LY. Willingness to Pay for a COVID‐19 Vaccine. *App Health Econ Health Policy* 2021;**19**:343-51. doi: 10.1007/s40258-021-00644-6

4. Pfizer. Global Vaccine Differential Pricing Approach, 2018. Available: https://cdn.pfizer.com/ pfizercom/health/vaccines/PFE_Global_Vaccines_Tiered_Pricing_Approach_03MAR2018.pdf [Accessed 16 Apr 2021].

5. GlaxoSmithKline. GSK Public policy positions, 2019. Available: https://www.gsk.com/media/5683/ tiered-pricing-and-vaccines-aug19.pdf [Accessed 16 Apr 2021].

6. Merck. Merck’s Position Statement – Pricing, 2019. Available: https://www.merckgroup.com/company

/who-we-are/en/healthcare/Pricing.pdf [Accessed 16 Apr 2021].

7. Sanofi. Access to Healthcare, 2020. Available: https://www.sanofi.com/-/media/Project/One-Sanofi-

Web/Websites/Global/Sanofi-COM/Home/common/docs/download-center/Access-to-Healthcare-2020.pdf?la=en [Accessed 16 Apr 2021].

8. Janssen. Janssen Access and Pricing Principles, 2021. Available: https://www.janssen.com/about/

access-pricing-principles [Accessed 16 Apr 2021].

9. [dataset] World Bank. Data from: GNI (current US$), 2021. Available: https://data.worldbank.org/ indicator/NY.GNP.MKTP.CD [Accessed 8 Jan 2021].

10. [dataset] World Bank. Data from: Population, total, 2021. Available: https://data.worldbank.org/ indicator/SP.POP.TOTL [Accessed 8 Jan 2021].

11. [dataset] World Bank. Data from: Poverty headcount ratio at $1.90 a day (2011 PPP) (% of population), 2021. Available: https://data.worldbank.org/indicator/SI.POV.DDAY [Accessed 8 Jan 2021].

12. [dataset] Institute for Health Metrics and Evaluation. Data from: Global Burden of Disease Study (GBD 2017), 2017. Available: http://ghdx.healthdata.org/gbd-2017 [Accessed 8 Jan 2021].

13. Gavi, the Vaccine Alliance. Gavi Alliance Eligibility and Transition Policy Version 3.0, 2018. Available: https://www.gavi.org/sites/default/files/document/gavi-eligibility-and-transition-policypdf.pdf [Accessed 8 Jan 2021].

14. World Health Organization. Immunization, Vaccines and Biologicals: Research and Product Development by Disease, 2021. Available: https://www.who.int/teams/immunization-vaccines-and-biologicals/diseases [Accessed 8 Jan 2021].

15. Plotkin S, Robinson JM, Cunningham G, *et al*. The complexity and cost of vaccine manufacturing – An overview. *Vaccine* 2017;**35**(33):4064–71. doi: 10.1016/j.vaccine.2017.06.003

16. Gouglas D, Le TT, Henderson K, *et al*. Estimating the cost of vaccine development against epidemic infectious diseases: a cost minimisation study. *Lancet Glob Health* 2018;**6**:e1386–96. doi: 10.1016/S2214-109X(18)30346-2

17. Wouters OJ, McKee M, Luyten J. Estimated Research and Development Investment Needed to Bring a New Medicine to Market, 2009-2018. *JAMA* 2020;**323**(9):844–53. doi: 10.1001/jama.2020.1166

18. Pronker ES, Weenen TC, Commandeur H, *et al*. Risk in Vaccine Research and Development Quantified. *PLoS One* 2013;**8**(3):e57755. doi: 10.1371/journal.pone. 0057755

19. Prasad V, Mailankody S. Research and Development Spending to Bring a Single Cancer Drug to Market and Revenues After Approval. *JAMA Intern Med* 2017;**177**(1):1569–75. doi: 10.1001/jamainternmed.2017.3601

20. LaMattina J. The Conundrum of Investing In Cardiovascular Versus Rare Diseases R&D. Forbes. Jan 9, 2020. Available: https://www.forbes.com/sites/johnlamattina/2020/01/09/the-conundrum-of-investing-in-cardiovascular-versus-rare-diseases-rd/?sh=1ac0b397 5f45 [Accessed 9 Feb 2021].

21. Biotechnology Innovation Organization, BioMedTracker, Amplion. Clinical Development Success Rates 2006-2015, 2016. Available: https://www.bio.org/sites/default/files/legacy/bioorg/docs/Clinical

%20Development%20Success%20Rates%202006-2015%20-%20BIO,%20Biomedtracker,%20

Amplion%202016.pdf [Accessed 9 Feb 2021].

22. Wong CH, Siah KW, Lo AW. Estimating Clinical Trial Success Rates and Related Parameters in Oncology. Working Paper. 2019; May 5. doi:10.2139/ssrn.3355022

23. Evaluate Ltd. EvaluatePharma World Preview 2019, Outlook to 2024, 2019. Available: https://info.evaluate.com/rs/607-YGS-364/images/EvaluatePharma_World_Preview_2019.pdf [Accessed 9 Feb 2021].

24. Fortune Business Insights. Cardiovascular Drugs Market, 2019. Available: https://www.fortune

businessinsights.com/industry-reports/cardiovascular-drugs-market-100379 [Accessed 9 Feb 2021].

25. World Health Organization. Global Vaccine Market Report, 2019. Available: https://apps.who.int/iris/ handle/10665/311278 [Accessed 8 Jan 2021].

26. World Bank. World Bank Country and Lending Groups, 2021. Available: https://datahelpdesk. worldbank.org/knowledgebase/articles/906519-world-bank-country-and-lending-groups [Accessed 8 Jan 2021].

27. Cernushi T, Gilchrist S, Hajizada A, *et al*. Price transparency is a step towards sustainable access in middle income countries. *BMJ* 2020;**368**:15375. doi: 10.1136/bmj.l5375

28. World Health Organization. Global Vaccine Market Report, 2020. Available: https://www.who.int/immunization/programmes_systems/procurement/mi4a/platform/module2/2020_Global_Vaccine_Market_Report.pdf?ua=1 [Accessed 8 Jan 2021].

29. André FE. How the research-based industry approaches vaccine development and establishes priorities. *Devel Biol* 2002;**110**:25–9.

30. Serdobova I, Kieny MP. Assembling a Global Vaccine Development Pipeline for Infectious Diseases in the Developing World. *Am J Public Health* 2006;**96**(9):1554–59. doi: 10.2105/AJPH.2005.074583

31. [dataset] Ruggles S, Flood S, Foster S, *et al*. Data from: IPUMS USA: Version 11.0. Minnesota: IPUMS, 2021. Available: https://doi.org/10.18128/D010.V11.0 [Accessed 5 Jan 2021].
